# Italian Adaptation and Psychometric Validation of the Cardiac Distress Inventory in Patients with Recent Cardiac Events

**DOI:** 10.3390/bs16091599

**Published:** 2026-09-08

**Authors:** Graziano Gigante, Edward Callus, Sara Gostoli, Enrico Giuseppe Bertoldo, Valentina Fiolo, Giulia Lorefice, Sofia De Stefano, Giulia Casu, Marco Monti, Stefano Urbinati, Alun Conrad Jackson, Chiara Rafanelli

**Affiliations:** 1 Department of Psychology “Renzo Canestrari”, University of Bologna, Viale Carlo Berti Pichat, 5, 40127 Bologna, Italy; graziano.gigante2@unibo.it (G.G.); sara.gostoli2@unibo.it (S.G.); giulia.casu3@unibo.it (G.C.); 2 Clinical Psychology Service, IRCCS Policlinico San Donato, Via Morandi 30, 20097 San Donato Milanese, Italy; edward.callus@grupposandonato.it (E.C.); enricogiuseppe.bertoldo@grupposandonato.it (E.G.B.); valentina.fiolo@grupposandonato.it (V.F.); giulia.lorefice@grupposandonato.it (G.L.); sofiadeste@hotmail.it (S.D.S.); 3 Department of Biomedical Sciences for Health, Università degli Studi di Milano, Via Mangiagalli 31, 20133 Milano, Italy; 4 Hospital Psychological Service, Bellaria Hospital, AUSL Bologna, Via Altura, 5, 40139 Bologna, Italy; marco.monti@ausl.bologna.it; 5 Division of Cardiology, Bellaria Hospital, AUSL Bologna, Via Altura, 5, 40139 Bologna, Italy; stefano.urbinati@ausl.bologna.it; 6 Australian Centre for Heart Health, The Royal Melbourne Hospital, P.O. Box 2137, Melbourne, VIC 3050, Australia; alun.jackson@australianhearthealth.org.au; 7 School of Psychological Sciences, University of Melbourne, Parkville, VIC 3010, Australia; 8 Centre on Behavioral Health, The Centennial Campus, University of Hong Kong, CJT-706, 7/F., The Jockey Club Tower, Pokfulam, Hong Kong

**Keywords:** cardiac distress inventory, cardiovascular disease, psychometrics, cultural adaptation

## Abstract

Background: Acute cardiac events trigger multifaceted psychological responses captured by the construct of cardiac distress, a persistent negative emotional state involving multiple psychosocial domains. The Cardiac Distress Inventory (CDI) assesses this construct, but no Italian version exists. This study aimed to translate the CDI and assess its psychometric properties. Methods: After translation and back-translation, face validity was assessed with five patients and content validity with healthcare professionals. The CDI was administered to 293 patients who experienced a recent cardiac event from two Italian centers. Missing data were handled with multiple imputations. Exploratory factor analysis (EFA; principal axis factoring, oblimin rotation) assessed factorial structure. Criterion validity was assessed using correlations with Emotion Thermometers, Patient Health Questionnaire-4 (PHQ-4) and Kessler-6 (K6); ROC analysis assessed discriminative accuracy and the optimal cut-off. Results: The questionnaire demonstrated good face validity and suboptimal content validity (CVI-S/Ave = 0.72). EFA revealed a five-factor structure explaining 54% of the variance: “Role Change and Adaptation to Disease”, “Emotional Overwhelm, “Cognitive Challenges”, “Social Disconnection”, and “Health System Challenges”. The final 46-item version showed good internal consistency (ω = 0.857–0.926) and discriminative accuracy (AUC = 0.84), with an optimal cut-off of 36 (sensitivity 71%, specificity 84%). Conclusions: The current study provides preliminary evidence for the validity of the Italian CDI. Additional studies are needed to confirm the proposed structures and clinical cut-offs.

## 1. Introduction

Cardiovascular diseases (CVD) represent a leading cause of mortality and morbidity worldwide (Roth et al., 2020). In Italy, prevalence is particularly high (12.87% in 2017), likely due to population aging, with CVD accounting for 34.81% of all deaths (Saglietto et al., 2021).

A relevant associated issue is the bidirectional relationship between CVDs and mental health (Bueno et al., 2025). Depression occurs in 15.0–31.3% of cardiovascular patients, while anxiety affects 27–32.9% (Karami et al., 2023; B. Murphy et al., 2019; Rafiei et al., 2023; Storer et al., 2023). Both conditions are associated with poorer prognosis (Bueno et al., 2025).

Beyond depression and anxiety, cardiac diseases and events can trigger diverse psychological responses, including post-traumatic stress disorder or symptoms (Bueno et al., 2025) and denial (Worcester, 2015), which serves as a coping mechanism in 3.0–53.2% of patients (Patierno et al., 2023). Patients may also experience grief reactions related to autonomy loss and identity changes (Higgins et al., 2007; Worcester, 2015), often accompanied by feelings of anger, guilt, and subclinical depressive symptoms, collectively referred to as ‘cardiac blues’ (Higgins et al., 2007; B. M. Murphy et al., 2015).

Contemporary research has increasingly focused on a comprehensive conceptualization of psychological experiences following cardiac events, leading to the *cardiac distress* construct: a persistent negative emotional state involving multiple psychosocial domains that challenges patients’ capacity to cope with their condition, treatment, daily living changes, sense of self, and future orientation. Cardiac distress encompasses previously identified psychological responses and additional components, including physical symptom perception, grief related to identity changes and functionality loss, fear of disease progression, recurrence, and death, sexual concerns, and sleep disturbances (Jackson et al., 2022b). The clinical relevance of cardiac distress assessment has been further underlined in the 2025 European Society of Cardiology Clinical Consensus Statement on Mental Health and Cardiovascular Disease (Bueno et al., 2025), which recommends its inclusion as a potential component of routine screening protocols for cardiovascular patients.

Recognizing the need for comprehensive psychological assessment in cardiac populations, Jackson et al. (2022a) developed the Cardiac Distress Inventory (CDI). This 55-item instrument evaluates the presence and severity of symptoms across eight subscales. A 12-item short form (CDI-SF; Le Grande et al., 2023) was subsequently developed. Cross-cultural translations of the CDI are available in Chinese (Fong et al., 2024), Farsi (Sharif-Nia et al., 2025b), and Turkish (Afşar et al., 2025).

In Italy, psychological evaluation of cardiac patients currently follows a fragmented approach. National guidelines (Consiglio Nazionale Ordine degli Psicologi, 2024) recommend the use of brief questionnaires, which are often not specifically designed for cardiac populations. Commonly used instruments include the Symptoms CheckList-90-R (Prunas et al., 2012) and the Beck Depression Inventory II (Ghisi et al., 2006). While some cardiac-specific instruments are available in Italian, such as the Surgical Fear Questionnaire (Damico et al., 2024) and the HeartQoL (Fattirolli et al., 2022), none comprehensively assess cardiac distress as conceptualized above.

This study aims to develop an Italian version of the CDI and evaluate its psychometric properties in a sample of patients with recent cardiac events. We hypothesize that the Italian CDI will show adequate validity and reliability for future comprehensive psychological assessment of cardiac patients across clinical and research applications.

## 2. Materials and Methods

### 2.1. Procedures

This study employed a sequential two-phase design: (1) translation and cross-cultural adaptation with preliminary validation, and (2) comprehensive psychometric testing in the target clinical population. Assessments were conducted by clinical psychologists with experience in cardiovascular psychology. Data collection followed standard confidentiality protocols, with prompt anonymization/pseudonymization and secure storage.

The study received ethical approval from the Ethics Committees of the local health authorities (Comitato Etico Area Vasta Emilia Centro, AUSL Bologna, Italy, Ref: 155-2023-OSS-AUSLBO, 16 February 2023; Comitato Etico dell’Ospedale San Raffaele, IRCCS Ospedale San Raffaele, Italy, Ref: 119/2023, 10 May 2023) and adhered to the ethical standards of the institutional and national research committees and to the Helsinki Declaration (World Medical Association, 2013).

The generative artificial intelligence models Claude Sonnet 4, Claude Sonnet 4.5, Claude Opus 4.1, Claude Opus 5 (Anthropic, San Francisco, CA, USA), and Grammarly v1.2.288.1945 ( Superhuman, San Francisco, CA, USA) were used in September 2025, October 2025, and August 2026 to aid with grammar revision and conceptual content review of the present manuscript. The authors reviewed all AI suggestions critically and maintain full responsibility for the scientific content, methodology, and conclusions.

This article is a revised and expanded version of a conference abstract entitled “Assessing cardiac distress in Italian patients: Translation and validation of the cardiac distress inventory, a comprehensive psychosomatic tool” (Gigante et al., 2026), which was presented at the 13th Annual Scientific Conference of the European Association for Psychosomatic Medicine (EAPM), Florence, 16–19 June 2026.

#### 2.1.1. Phase 1: Translation and Item Pre-Testing

The translation process was authorized by the original authors and followed established cross-cultural adaptation guidelines (Beaton et al., 2000). Forward translation was performed by a bilingual healthcare professional, followed by back-translation by a native English speaker fluent in Italian and blinded to the original questionnaire content. Translators focused on equivalence, simplicity, and culturally appropriate language. Discrepancies were resolved through a consensus meeting with the research team.

Face validity was assessed through individual interviews with five patients from the target population to evaluate item comprehensibility, acceptability, and emotional impact.

Content validity was evaluated by 11 cardiac healthcare professionals using a four-point scale according to Lynn’s guidelines (1986).

#### 2.1.2. Phase 2: Item Testing

Recruitment occurred between March 2023 and November 2024 at two Italian centers. The Cardiac Division of Bellaria Hospital (Bologna) recruited consecutive outpatients undergoing cardiac rehabilitation, whereas the Heart Surgery Division of IRCCS Policlinico San Donato (San Donato Milanese) recruited cardiac surgery patients before discharge.

After a comprehensive explanation of the study and informed consent procedures, participants completed questionnaires either on-site or at home, with quality data checks conducted upon return. The assessment took around 35 min and began with the collection of sociodemographic and clinical characteristics through a structured form, verified through patient interviews and medical records. The primary measure was the Italian version of the CDI. Secondary measures included: Emotion Thermometers (ET) (Mitchell et al., 2012), Kessler Psychological Distress Scale-6 (K6) (Carrà et al., 2011), and Patient Health Questionnaire-4 (PHQ-4) (Kroenke et al., 2009). Additionally, the allostatic overload section of the Diagnostic Criteria for Psychosomatic Research Semi-Structured Interview (DCPR-SSI) (Fava et al., 2017), a single item from the PsychoSocial Index (PSI) related to quality of life (Piolanti et al., 2016), and the Morisky Medication Adherence Scale-8 (MMAS-8) (Fabbrini et al., 2013) were administered but not used in the present study. Additional clinical data, including pharmacological treatments and physiological parameters, were extracted from medical records for a participant subsample. The current analysis focuses on core psychometric validation measures.

### 2.2. Participants

Adults who had experienced a cardiac event (e.g., myocardial infarction, bypass surgery, valve surgery, arrhythmia requiring intervention) within the previous 12 months were considered eligible. Exclusion criteria included inadequate Italian language proficiency or any condition that would impair comprehension of the informed consent process or study instruments.

Sample size followed the guideline of five participants per item (55 items × 5 = 275 participants), providing sufficient power for Exploratory Factor Analysis (EFA) (Bryant & Yarnold, 1995). Recruitment aimed for at least 280 participants for site balance. Sample size adequacy was further assessed post hoc using Mundfrom et al.’s (2005) criteria, based on communalities, the average number of variables per factor, and the total number of factors in the solution.

### 2.3. Measures

#### 2.3.1. Sociodemographic and Clinical Data

Sociodemographic and clinical data were collected using a modified version of the instrument utilized in the original CDI validation study (Jackson et al., 2022a). Variables included age, gender, marital status, living arrangements, employment status, education, financial difficulties, type of cardiac event, comorbidities, and participation in cardiac rehabilitation programs.

#### 2.3.2. Cardiac Distress Inventory (Jackson et al., 2022a)

The CDI comprises 55 items describing post-cardiac event experiences. Respondents indicate whether each item was experienced and rate associated distress on a 4-point scale (0 = none, 3 = severe). Items not experienced are scored as 0. The total instrument score ranges from 0 to 165 across 8 subscales representing core psychosocial domains relevant to cardiac populations. Established cut-offs classify moderate (≥25) and severe (≥37) cardiac-related emotional distress. The original CDI demonstrated adequate to good internal consistency across all subscales (Cronbach’s α = 0.73–0.87) and satisfactory criterion validity (Jackson et al., 2022a).

#### 2.3.3. Emotion Thermometers (ETs) (Mitchell et al., 2012)

The ETs include four 0–10 visual analog scales assessing distress, anxiety, depression, and anger over the past week. These instruments have demonstrated good criterion validity and reliability in oncological populations (Harju et al., 2019) and good clinical sensitivity for detecting psychological distress in cardiovascular patient populations (Mitchell et al., 2012). A separate thermometer assessed COVID-19-related anxiety or worry over the past week to distinguish between distress attributable to the ongoing COVID-19 pandemic and distress specifically related to participants’ cardiac conditions, consistent with the original CDI validation (Jackson et al., 2022a).

#### 2.3.4. Kessler Psychological Distress Scale-6 (K6) (Carrà et al., 2011)

The K6 is a 6-item psychological distress screening instrument validated in Italian populations. Score ranges from 0 to 24, with higher scores indicating greater distress. The Italian version demonstrated excellent internal consistency (Cronbach’s α = 0.84) and good discriminative accuracy for distinguishing between individuals with and without severe mental health conditions (Carrà et al., 2011). In this study, the K6 exhibited good internal consistency (McDonald’s ω = 0.84).

#### 2.3.5. Patient Health Questionnaire-4 (PHQ-4) (Kroenke et al., 2009)

The PHQ-4 is a screening tool consisting of 4 items that assess psychological distress. The total score can range from 0 to 12, based on two subscales, each containing 2 items, that measure anxiety and depression. The PHQ-4 has been identified as a recommended screening tool for anxiety and depression in cardiovascular contexts (Bueno et al., 2025; Ski et al., 2025), and specific cut-offs for anxiety and depression have been developed for this purpose in the Italian context (Giuliani et al., 2021). However, there is a lack of proper validation for the cut-off scores of the total score in the Italian context. In this study, the PHQ-4 showed good internal consistency, with a McDonald’s ω of 0.84.

### 2.4. Data Analysis

Missing data analyses and imputation were performed using SPSS Statistics for Windows, version 29.0.2.0 (IBM, Armonk, NY, USA); content validity analyses were conducted in Microsoft Excel for Windows, version 2508 (Microsoft, Redmond, WA, USA); all remaining analyses were conducted using R (v4.4.3; R Core Team, 2025) on RStudio (v2026.7.1.147; Posit Team, 2026; Boston, MA, USA). Statistical analysis scripts for R were generated using Claude Opus 5 (Anthropic, San Francisco, CA, USA) in August 2026 and subsequently reviewed and edited by the authors. Descriptive statistics summarized sample characteristics. Distribution normality was assessed visually and with the Shapiro–Wilk test when appropriate.

#### 2.4.1. Pre-Testing Analysis

Content validity indices and modified kappa coefficients were calculated according to Lynn’s guidelines (1986). Expert ratings of item relevance were first dichotomized to create binary relevance judgments: ratings of 1 (“Irrelevant”) and 2 (“Somewhat relevant”) were classified as “Not relevant,” while ratings of 3 (“Quite relevant”) and 4 (“Highly relevant”) were classified as “Relevant.” This dichotomization enabled the calculation of the content validity index (CVI) at the item level (CVI-I), which is the proportion of experts who rated each item as relevant. To account for chance agreement among experts, modified kappa coefficients were calculated for each item (Wynd et al., 2003). Kappa values were interpreted using established criteria: poor (κ < 0.40), fair (κ ≥ 0.40), good (κ ≥ 0.60), or excellent (κ ≥ 0.75) content validity (Cicchetti & Sparrow, 1981). Content validity at the subscale and total scale levels was evaluated using CVI-S/Ave, the average of CVI-I across items in a subscale; values ≥ 0.79 were considered adequate (Lynn, 1986).

Missing data analysis was performed as a preliminary step before psychometric evaluation, examining CDI, K6, PHQ-4, ET, and COVID-19 concern items. Little’s MCAR test was used to assess the missing data mechanism across all items. Given the low proportion of missing data and the failure to reject the Missing Completely at Random (MCAR) hypothesis, multiple imputations were employed to handle missing values. Five multiply imputed datasets were generated using fully conditional specification with predictive mean matching to accommodate the non-normal distribution of continuous variables. The imputation model included site of recruitment, demographic variables, clinical characteristics, quality of life, and all available CDI, K6, and Emotion Thermometer items as predictors. Post-imputation diagnostics confirmed distributional and structural similarity between observed and imputed values. Analyses were conducted separately for each imputed dataset, with results pooled using Rubin’s combination rules when possible (Rubin, 2018). The complete-case and imputed solutions were compared to assess the robustness of the factor structure to the treatment of missing data, examining factor composition, communalities, explained variance, reliability, and Tucker congruence coefficient (Tucker, 1951). Still, given the modest sample size of the complete cases (*n* = 277) and the statistical advantages of multiple imputation (van Buuren, 2018), only imputed results are presented in the main text. Imputed datasets were subsequently imported into R and managed using the R package mice (v3.17.0; van Buuren & Groothuis-Oudshoorn, 2011).

#### 2.4.2. Exploratory Factor Analysis

An Exploratory Factor Analysis (EFA) was conducted using a polychoric correlation matrix to identify the questionnaire’s underlying factor structure (Goretzko et al., 2021). Polychoric correlations are recommended for ordinal data with fewer than five response categories or with skewed frequency distributions (Holgado-Tello et al., 2010). No continuity correction was applied to zero-frequency cells, following the recommendation for items with more than two response categories (Savalei, 2011). If the resulting matrix was not positive definite, it was smoothed to the nearest positive definite matrix (Higham, 2002) prior to factoring (Debelak & Tran, 2016; Lorenzo-Seva & Ferrando, 2021).

Data adequacy was confirmed through Kaiser–Meyer–Olkin sampling adequacy (KMO ≥ 0.60; Kaiser & Rice, 1974). Bartlett’s test (Bartlett, 1950) is reported for completeness, acknowledging its sensitivity to sample size and its reliance on assumptions that do not strictly hold for polychoric matrices.

Factor retention was based on parallel analysis (PA; Horn, 1965) and Velicer’s minimum average partial procedure (MAP; Velicer, 1976). Parallel analysis was conducted with 500 permutations of the observed data (Buja & Eyuboglu, 1992); factors were retained when observed eigenvalues exceeded the 95th percentile of the permuted distribution (Glorfeld, 1995). Eigenvalues were computed from the reduced correlation matrix with squared multiple correlations on the diagonal, consistent with the common factor model underlying principal axis factoring; this variant has been shown to outperform the principal-component implementation when item complexity is high or a general factor is present (Crawford et al., 2010). Both criteria were computed separately in each of the five imputed datasets and summarised by their modal value, as no consensus exists on pooling rules for factor-retention indices. Incongruencies between PA and MAP results were reported and compared: the final number of retained factors was determined by ensuring at least three items per factor, acceptable reliability (ω > 0.70), a minimal number of cross-loadings (items with relevant loading on multiple factors, with a difference in loadings < 0.10; Güvendir & Özkan, 2022; Watkins, 2018), and theoretical coherence (Watkins, 2018). Scree plots and eigenvalues are reported for descriptive purposes.

Factors were extracted by principal axis factoring, with direct oblimin rotation (δ = 0) and Kaiser normalization to allow for correlated factors.

Items were evaluated for removal through three iterative steps, continuing until all removal or decision criteria were addressed:

1. Items with factor loadings < 0.32 were systematically removed. The threshold was defined consistently with established guidelines (Tabachnick & Fidell, 2014), similarly to the original validation study (Jackson et al., 2022a), and confirmed as adequate by Norman and Streiner’s formula, indicating a minimum suggested loading of 0.302 for the sample size of the present study. An item was removed only if it fell below the threshold in all five imputed datasets, ensuring that the retained set of items remained identical across the imputations. The EFA was then conducted again on the new solution to evaluate its stability. This step was repeated until no additional items were identified as falling below the threshold.
2. Items with loadings of 0.32 or higher on multiple factors, with less than a 0.10 difference between those loadings, were classified as cross-loadings. Each of these items was assessed for theoretical relevance on both factors, beginning with the item that showed the smallest difference overall. If an item was deemed irrelevant, it was removed, and the entire procedure was repeated from the first step after each removal. (Güvendir & Özkan, 2022).
3. Finally, items showing a corrected item–total correlation (ITC) below 0.30 (Cristobal et al., 2007), computed with each item excluded from its own subscale total, together with low theoretical relevance or content redundancy with other retained items, were considered for removal, beginning with the lowest ITC. An item was removed only where its deletion did not decrease subscale omega by more than 0.02 (Xiao et al., 2024). After each removal, the whole procedure was repeated from the first step.

#### 2.4.3. Bifactor and Higher-Order Representations

To evaluate the extent to which a general factor accounted for the common variance among items, and whether the retained group factors contributed reliable variance beyond it, the correlated-factors solution was examined in hierarchical and bifactor form. A second-order factor was extracted from the inter-factor correlation matrix by principal axis factoring, and this hierarchical solution was then orthogonalized using the Schmid–Leiman transformation (Schmid & Leiman, 1957) to obtain an approximate bifactor representation. From the orthogonalized solution, omega hierarchical (ω*_H_*), omega total (ω*_t_*), subscale omega hierarchical (ω*_HS_*), subscale omega total (ω*_S_*), construct replicability (H), and the explained common variance (ECV) were estimated as described by Rodriguez et al. (2016) using the R package BifactorIndicesCalculator (v0.2.2; Dueber, 2021). Values of ω*_H_* ≥ 0.80, together with ECV ≥ 0.70, were taken to indicate that the scale was essentially unidimensional and that a total score could be meaningfully interpreted; ω*_HS_* was interpreted relative to ω*_S_* as the proportion of reliable subscale variance independent of the general factor; values of H ≥ 0.80 determined an adequate construct replicability (Rodriguez et al., 2016). Consistent with Rodriguez et al. (2016), these values were treated as interpretive heuristics rather than as decision rules.

EFA-related analyses were conducted using the R packages psych (v2.5.3; Revelle, 2025) and Matrix (v1.7-2; Bates et al., 2025).

#### 2.4.4. Reliability

Reliability was assessed for each factor and for the total scale using McDonald’s omega (McDonald, 1999) computed from the polychoric correlation matrix (ω > 0.70 acceptable, >0.80 good, >0.90 excellent). Reliability coefficients were computed using the R package psych (v2.5.3; Revelle, 2025).

#### 2.4.5. Concurrent and Discriminant Validity

Concurrent validity was assessed through Spearman’s correlations between CDI and established distress measures (Distress Thermometer, K6, PHQ-4). Discriminant validity was evaluated by examining correlations between the CDI and theoretically unrelated constructs (COVID-19 concern, Anger Thermometer). Correlations were computed within each imputed dataset, Fisher-transformed, pooled using Rubin’s rules, and back-transformed. The transformation was applied because Rubin’s rules assume approximate normality of the pooled quantity, which a correlation coefficient does not have; applied to Spearman coefficients, it remains an approximation, as the transformation was derived for Pearson correlations (van Buuren, 2018).

#### 2.4.6. Discriminative Accuracy Analysis and Cut-Off Determination

Receiver operating characteristic (ROC) curve analysis was conducted to find the optimal CDI cut-off score for high distress potentially associated with mental illness, using the K6 cut-off (≥7; Carrà et al., 2011) as a criterion. While the K6 is not specifically designed to measure cardiac distress, there is currently no gold standard for this recently identified construct. Therefore, K6, as a general measure of distress, could serve as a useful comparison. In the absence of a gold standard, ROC curve analyses were conducted using the PHQ-4 cut-off and the Distress Thermometer cut-off (≥5; Grassi et al., 2013) for post hoc sensitivity purposes. Due to the lack of an Italian-validated cut-off for the PHQ-4 and given the sensitivity purpose of the current analysis, an international cut-off of 6 was used (Larionow & Karolina Mudło-Głagolska, 2023; Löwe et al., 2010; Obeid et al., 2024). The optimal cut-off was determined using Youden’s index (Schisterman et al., 2005). Discriminative accuracy was assessed through area under the curve (AUC) values, interpreted as acceptable (>0.70), good (>0.80), or excellent (>0.90) (Çorbacıoğlu & Aksel, 2023). The same K6-based procedure was applied to determine screening cut-offs for each subscale, identifying levels of general distress associated with each domain. AUC values were pooled across imputed datasets using Rubin’s rules applied to logit-transformed estimates, as Rubin’s rules assume approximate normality of the pooled quantity and the AUC is bounded between 0 and 1 (van Buuren, 2018). ROC analyses were performed using the R package pROC (v1.18.5; Robin et al., 2011); figures were produced with ggplot2 (v3.5.2; Wickham, 2016).

#### 2.4.7. Exploratory Analyses

Given the heterogeneity of the sample, an exploratory multiple linear regression model was developed to examine whether recruitment site, time since the cardiac event, and type of cardiac event were associated with the total CDI score computed from the final solution. Because patients could report more than one cardiac event, event types were entered as separate binary indicators (1 = present, 0 = absent). Event types reported by more than ten participants were entered as separate binary indicators; the remaining types were included in the pre-existing “Other” category. Attendance at cardiac rehabilitation was excluded from the model because it was collinear with recruitment site (VIF = 11.5 and 12.5, respectively), as the two centers differed systematically in patients’ phases of cardiac disease treatment (before or during rehabilitation). The model was estimated separately in each of the five imputed datasets and pooled using Rubin’s rules. Normality of residuals, homoscedasticity, and multicollinearity (Variance Inflation Factor; VIF) were examined. Unstandardized coefficients with 95% confidence intervals, *p*-values, and the model *R*^2^ are reported, with *p* < 0.05 used as a conventional threshold. Given the exploratory nature of the analysis, no correction for multiple comparisons was applied.

## 3. Results

### 3.1. Phase 1: Item Pre-Testing

#### 3.1.1. Face Validity

Face validity interviews were conducted with five cardiac patients (four men, one woman; median age = 76 years, *IQR* = 54–85). Completion required a median of 15 min (IQR = 10–20). Participants had experienced various recent cardiac events: heart valve issues, heart failure, percutaneous coronary intervention, coronary artery bypass graft surgery, and heart rhythm disturbances. Patient feedback revealed that four participants found the questionnaire clear and understandable, while two found it too long, and two found the items and responses too generic. One patient noted that some items did not reflect his situation, whereas another found them highly relevant to their experience. Comprehension difficulties were identified for items 1 (“Being physically restricted”), 10 (“Thinking about dying”), 47 (“Dwelling on my heart condition”), and 54 (“Being emotionally exhausted”). Following research team discussion, items 47 and 54 were reformulated for clarity: “Pensare costantemente alla mia situazione cardiaca” and “Essere emotivamente provato/a,” respectively. Items 1 and 10 were not reformulated because no alternative wording was found to express them more clearly.

#### 3.1.2. Content Validity

Modified Kappa coefficients were not normally distributed (*W* = 0.89, *p* < 0.001), ranging from 0.21 to 1.00 (*Mdn* = 0.70, *IQR* = 0.57–0.81); 26 items (47%) demonstrated excellent content validity (κ ≥ 0.75), 11 good (κ ≥ 0.60), 7 fair (κ ≥ 0.40), and 11 poor (κ < 0.40). Overall CVI-S/Ave was 0.72, with only Physical Challenges achieving adequate validity (CVI-S/Ave = 0.81). Despite suboptimal indices, all items were retained for empirical testing to maintain comparability with the original instrument and enable cross-cultural comparisons. Final item retention decisions were therefore deferred to exploratory factor analysis. Complete modified Kappa coefficients, CVI-I values, and subscale CVI-S/Ave scores are presented in Table 1.

### 3.2. Phase 2: Item Testing

#### 3.2.1. Sample Characteristics

At Bellaria Hospital, 153 patients (70.5% of 217 approached) were enrolled. Although the original target for this site was 140 patients, the time lag between informed consent and questionnaire completion led to the inclusion of 13 additional patients. At IRCCS Policlinico San Donato, 140 patients (~90% of 155 approached) were enrolled. The total sample included 293 participants.

Patient age was non-normally distributed (*p* < 0.001), with a median of 67 years (*IQR* = 59–74). CCI scores and number of comorbidities were also non-normally distributed (*p* < 0.001), with medians of 0 (*IQR* = 0–1), corresponding to a median 1-year in-hospital mortality rate of 0.9% in the tested European countries (Quan et al., 2011). Time since the most recent cardiac event was also non-normally distributed (*p* < 0.001), with a median of 17 days (*IQR* = 5–39). Complete sociodemographic and clinical characteristics are presented in Table 2 and Table 3.

#### 3.2.2. Missing Data Analysis

93.5% of participants (*n* = 274) completed all questionnaires entirely. Only 5.4% of the participants (*n* = 16) partially completed the CDI, 1.0% (*n* = 3) partially completed the K6, and 1.0% (*n* = 3) omitted at least one thermometer. All participants completed the PHQ-4. Missingness ratio for each item can be found in Table S1. Little’s Missing Completely at Random (MCAR) Test failed to reject the MCAR hypothesis, χ^2^(1127) = 1234.17, *p* = 0.607. However, multivariate normality was not confirmed; therefore, these results should be interpreted cautiously.

#### 3.2.3. Exploratory Factor Analysis

Given the stability of the number of retained factors (equivalence across imputations), the close similarity of loadings (maximum absolute difference = 0.024; Tucker’s congruence coefficients = 1.00), and the convergence of item removal decisions across imputed datasets, only the results from the first dataset are reported in the main text. Detailed results for the five datasets are provided in Tables S2–S11.

Data suitability was confirmed (KMO = 0.65; Bartlett’s test χ^2^(1485) = 65,399.3, *p* < 0.001). Both indices were computed on the smoothed polychoric matrix, since neither can be obtained from a matrix that is not positive definite, and are therefore reported as descriptive.

**Figure 1:**
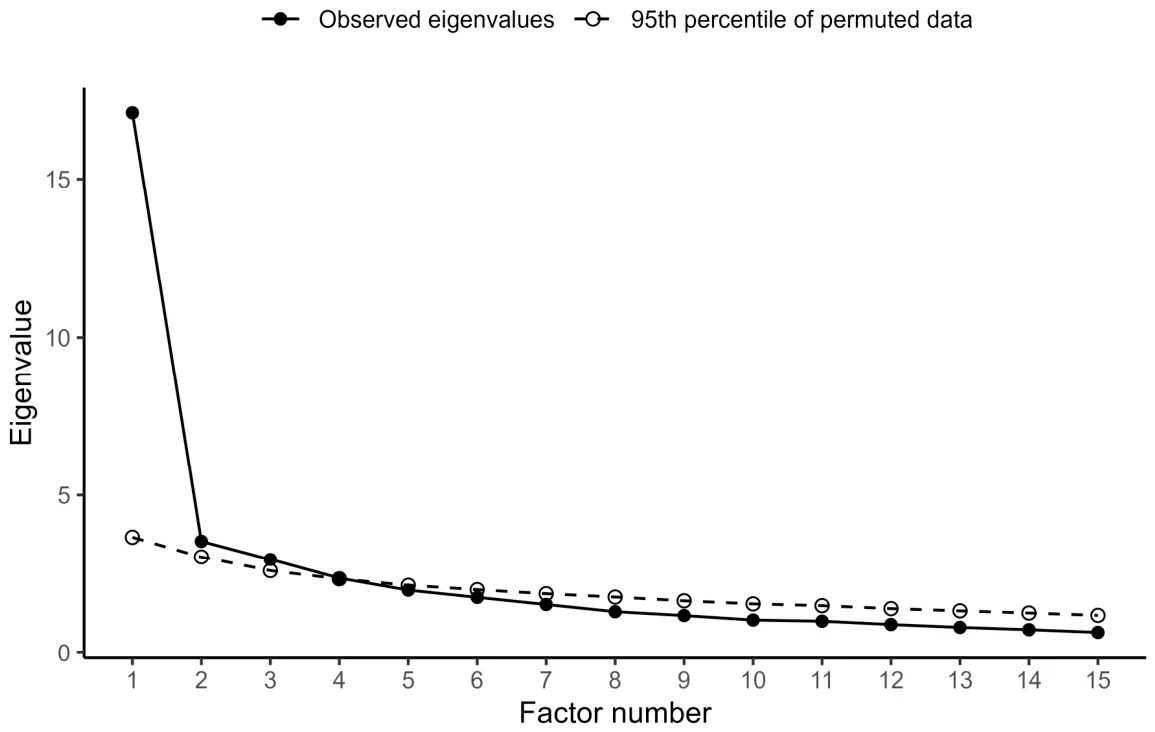
Scree plot comparing observed eigenvalues with the 95th percentile of 500 column-wise permutations of the observed data, computed on the initial 55-item set. Imputed data (*n* = 293), first imputed dataset.

PA indicated a suggested number of factors of 4 (Figure 1), while the MAP method suggested 5. Both solutions were analyzed and compared according to prespecified criteria. Eigenvalues of the factorial solution and the simulated PA dataset across imputations are reported in Table S3 for reference.

The five-factor solution underwent a 13-step decision process that resulted in the removal of eight items due to low loadings, removal of one item for low ITC and low theoretical relevance, and reassignment of a cross-loading item based on theoretical considerations. The final solution comprised 46 items that explained 54.0% of the total variance after rotation. The step-by-step decision process is reported in Supplementary Table S4.

Individual factor variance percentages were, respectively, 16.4% (Factor 1), 11.6% (Factor 2), 9.6% (Factor 3), 8.8% (Factor 4), and 7.5% (Factor 5). Communalities ranged from 0.26 to 0.86 (*M* = 0.54; *SD* = 0.14) and are reported in Table 4. Factor loadings and compositions are reported from the pattern matrix for ease of interpretation in Table 5. Factor correlation analysis showed low-to-moderate intercorrelations (φ = 0.080 to 0.384; Table 6). McDonald’s ω for the single factors were, respectively, 0.926 (Factor 1), 0.894 (Factor 2), 0.886 (Factor 3), 0.909 (Factor 4), and 0.857 (Factor 5).

The four-factor solution involved a 14-step decision-making process. This led to the removal of seven items because their loadings fell below the threshold. Additionally, one item was eliminated due to low ITC with no theoretical justification for its retention. A cross-loading item was also removed for lack of theoretical support, while two other cross-loading items were reassigned based on theoretical considerations. The final solution comprised 46 items that explained 48.8% of the total variance after rotation. The step-by-step decision process is reported in Table S12.

The individual factor variance percentages were 17.5% (Factor 1), 12.0% (Factor 2), 10.3% (Factor 3), and 9% (Factor 4). Communalities ranged from 0.24 to 0.76 (*M* = 0.49; *SD* = 0.11) and are reported in Table S13. Factor loadings and compositions are reported from the pattern matrix for ease of interpretation in Table S14. Factor correlation analysis showed low-to-moderate intercorrelations (φ = 0.129 to 0.400; Table S15). The solution exhibited excellent reliability (ω = 0.95), with McDonald’s ω for the single factors of 0.926 (Factor 1), 0.891 (Factor 2), 0.882 (Factor 3), and 0.878 (Factor 4).

Both solutions met the pre-specified selection criteria concerning the minimum number of items per factor, reliability, and a low number of cross-loadings. Neither presented any factors with fewer than three items or factors with ω ≤ 0.70. Additionally, the number of cross-loaded items was low in both solutions, with three items in the four-factor solution and four in the five-factor solution. In terms of theoretical coherence, all items in the five-factor solution showed coherence with their assigned factor, except item 55, “Lacking purpose or meaning in life”, which was theoretically poorly related to the other items in its factor. In the 4-factor solutions, item 52 (Being short of breath) was completely unrelated to the other items of its factor. In the same vein, the five-factor solution had the strongest theoretical foundation, as it more closely matched the structure of the original questionnaire (Jackson et al., 2022a) by replicating two distinct factors: social disconnection and health system challenges. Although these two factors were included in the four-factor solution, they were grouped into a single factor, which might indicate a shared symptom of underfactoring (Watkins, 2018). Considering these points, the five-factor solution was chosen for further analysis.

Post hoc sample size assessment for the chosen solution indicated an adequate size, based on the minimum sample size criteria determined by Mundfrom et al. (2005). More specifically, a number of factors equal to five, a variable-to-factor ratio of 9.2 (46:5), and a wide distribution of communalities (0.26–0.86) are associated in their simulated dataset with a minimum sample size of 90 subjects.

MAP and PA analyses conducted on the complete-case dataset for comparison both indicated four factors for retention. The five-factor solution was nonetheless estimated for comparability with the primary analysis. The two solutions, compared on the final solution of 46 items, showed Tucker congruence coefficients ranging from 0.98 to 1.00 across the five factors, indicating substantial equivalence of the corresponding factors (Lorenzo-Seva & ten Berge, 2006), with comparable explained variance (53.8%) and reliability (subscales ω = 0.846–0.927; scale ω = 0.955). In addition to comparing the 46 shared items, both procedures underwent item selection, resulting in slight differences in the final sets of retained items. The complete-case analysis kept two items, specifically items 4 and 52, which had loadings within 0.02 of the salience threshold in the imputed datasets, while the primary analysis removed them. This resulted in a total of 48 items in the complete-case solution. Full results for the 48-item, 5-factor, complete-case solution can be found in Supplementary Materials (Tables S2, S3 and S16–S18).

#### 3.2.4. Bifactor and Higher-Order Representations

Results are reported for the first imputed dataset; values from the remaining four are provided in Tables S19–S25, as they showed high similarity. Model-level indices differed by no more than 0.001 across datasets, whereas factor-level indices showed slightly greater variability (maximum range 0.033).

Second-order loadings of the five factors on the second-order factor were, respectively, 0.654, 0.535, 0.485, 0.455, and 0.273, accounting for 24.6% of the variance. Schmid-Leiman loadings for all items on the general and group factors throughout imputations are reported in Tables S19–S23. Non-homogeneous distribution of second-order loadings across factors could indicate a violation of the proportional constraints required by the Schmid-Leiman transformation. Therefore, the present results should be considered approximations.

At the model level, omega hierarchical was 0.591, and the explained common variance was 0.356, both below the adopted thresholds of 0.80 and 0.70. Total omega was 0.968. Construct replicability for the general factor was above the 0.80 threshold (H = 0.918).

At the factor level, subscale omega hierarchical ranged from 0.349 to 0.434, compared with subscale omega total values of 0.915 to 0.942. Construct replicability exceeded 0.80 for four of the five group factors (H = 0.817–0.840), with Factor 4 below threshold (H = 0.760). Complete factor-level indices are reported in Table 7.

#### 3.2.5. Factor Compositions and Reliability Analysis

Results are reported for the first imputed dataset; values from the remaining four are provided in Table S26 as they showed high similarity (maximum difference = 0.002). McDonald’s ω indicated excellent internal consistency (ω = 0.952). Factor compositions and reliabilities are presented below. Final Item–total correlations and variations in McDonald’s omega for each factor after deletion of single items are reported in Table S27.

##### Factor 1: Role Change and Adaptation to Disease

Factor 1 contained 21 items related to changes in identity, plans, and activities, and perception of burdensome cardiac symptoms. It was named “Role change and adaptation to disease. The factor showed excellent reliability (ω = 0.926).

##### Factor 2: Emotional Overwhelm

Factor 2 contained ten items related to death, disease thoughts, and intense emotional responses, and it was therefore named “Emotional Overwhelm”. The factor showed good reliability (ω = 0.894).

##### Factor 3: Cognitive Challenges

Factor 3 contained five items related to cognitive problems, retaining therefore the original name, “Cognitive challenges”. The factor showed good reliability (ω = 0.886).

##### Factor 4: Social Disconnection

Factor 4 included six items related to social disconnection and loneliness, and it was named “Social disconnection”. The factor showed excellent reliability (ω = 0.909).

##### Factor 5: Health System Challenges

Factor 5 contained four items related to communication difficulties and unmet needs related to healthcare, and it was therefore named “Health System Challenges”, in accordance with the original subscale. The factor showed good reliability (ω = 0.857). 

#### 3.2.6. Concurrent and Discriminant Validity

The CDI demonstrated good concurrent validity, correlating moderately with the Distress Thermometer (ρ = 0.56, 95% CI = 0.47–0.63, *p* < 0.001), K6 (ρ = 0.43, 95% CI = 0.33–0.52, *p* < 0.001), and PHQ-4 (ρ = 0.69, 95% CI = 0.63–0.75, *p* < 0.001). Discriminant validity showed mixed results: the CDI correlated weakly with COVID-19 concern (ρ = 0.18, 95% CI = 0.06–0.29, *p* = 0.003) but moderately with anger (ρ = 0.53, 95% CI = 0.44–0.60, *p* < 0.001).

#### 3.2.7. ROC Analysis and Cut-Off Determination

Given equivalent results across datasets for the K6 criterion (fraction of missing information, fmi = 0.000–0.016, ΔYouden’s *J* = 0.000–0.024), only the findings from the first dataset are reported here, with detailed results for the remaining datasets provided in Tables S28 and S29.

Positive cases identified by the criteria were, respectively, *n* = 41 (14.0%) for K6, *n* = 68 (23.2%) for PHQ-4, and *n* = 134 (45.7%) for the Distress Thermometer (although the first imputation for the Distress Thermometer presented *n* = 135 cases, 46.1%).

**Figure 2:**
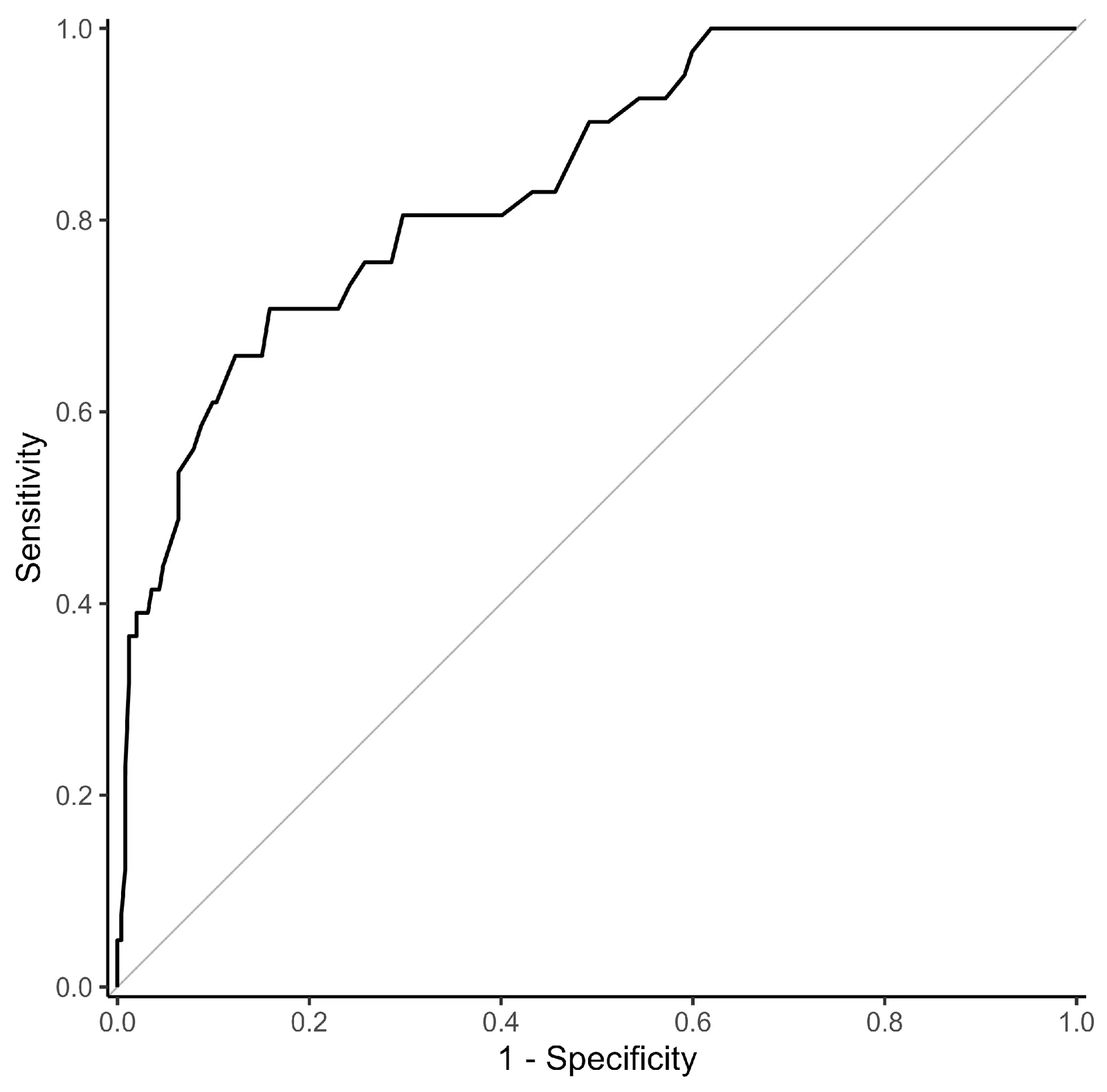
ROC Curve for CDI Total Score using Kessler-6 as Criterion Standard. The present figures refer to imputation dataset number 1.

ROC curve analysis with K6 as the criterion showed good discriminative accuracy (AUC = 0.84; 95% CI = 0.77–0.90) for identifying severe psychological distress potentially indicative of mental disorder (Figure 2). Using Youden’s index (0.549), the optimal CDI cut-off was identified as ≥36 (sensitivity = 71%; specificity = 84%).

ROC curve sensitivity analyses with PHQ-4 and the Distress Thermometer as criteria showed acceptable-to-good discriminative accuracy (AUCs of 0.84 and 0.76, respectively). Using Youden’s index, the optimal CDI cut-offs, though, were quite variable, with the one from PHQ-4 identified as ≥24 (Youden’s index = 0.552, sensitivity = 82%, specificity = 73%) or ≥23 (Youden’s index =0.551, sensitivity = 85%, specificity = 70%, imputation 2 and 3), and the one from the Distress Thermometer identified as ≥21 (Youden’s index = 0.400, sensitivity = 70%, specificity = 70%). A comparison between the different ROC curves is presented in Figure S1. Using the same K6-based criterion, ROC analyses for subscales yielded cut-offs for screening general psychological distress associated with each domain (Table 8). All subscale ROC curves are presented in Figures S2–S6.

#### 3.2.8. Exploratory Analyses

Pooled results presented a low fraction of missing information across coefficients (0.007), indicating that the imputation contributed little to the uncertainty of the estimates. The model accounted for a small proportion of variance in total CDI scores (adjusted *R*^2^ = 0.062, 95% CI = 0.019–0.125) and is reported in Table 9. The recruitment site showed a substantial association: participants recruited at Milano scored, on average, 7.71 points higher than those recruited at Bologna. Time since the cardiac event was not associated with total scores. Among event types, myocardial infarction was associated with higher scores, whereas coronary artery bypass grafting (CABG) was associated with lower scores. Model diagnostics indicated no collinearity among the retained predictors, but the residuals departed from normality and showed mild heteroscedasticity, with residual variance increasing across the range of fitted values. Confidence intervals should therefore be interpreted with caution.

## 4. Discussion

The purposes of the present study were to translate and assess the psychometric properties of the CDI in an Italian population with recent cardiac events. The Italian version of the CDI comprises 46 items across 5 subscales, differing from the original version. Furthermore, despite suboptimal content validity and mixed results on discriminant validity, it demonstrated good face validity, concurrent validity, reliability, and discriminant accuracy. The questionnaire and relative scoring methods are available in the Supplementary Materials.

### 4.1. Face and Content Validity

Face validity assessment led to revision of two items, with patients reporting clarity and comprehensibility, although the questionnaire was perceived as lengthy. While specific results regarding face validity are not reported in the original study (Jackson et al., 2022a), similar findings for clarity and comprehensibility are reported in the Farsi translation study (Sharif-Nia et al., 2025b).

Content validity analysis yielded unexpected results: 26 items were identified as candidates for removal, and seven of eight subscales, as well as the whole questionnaire, showed subthreshold content validity. This may reflect difficulties in understanding the tool or differences in how the panel interpreted the construct, but it may also indicate that the selected items and domains do not adequately represent cardiac distress in the Italian context. All items were nonetheless retained to enable cross-cultural comparisons, and the low content validity remains a relevant limitation of the instrument. Unfortunately, other cross-cultural CDI validations lack content validity assessment results, precluding comparison (Afşar et al., 2025; Fong et al., 2024; Sharif-Nia et al., 2025b).

### 4.2. Exploratory Factor Analysis Results

EFA led to the removal of nine items (16% of the original), identifying five main factors: “Role Change and Adaptation to Disease”, “Emotional Overwhelm”, “Cognitive Challenges”, “Social Disconnection”, and “Health System Challenges”. All removed items are reported in Supplementary Materials. The 46-item Italian CDI demonstrated excellent overall reliability, with the five subscales presenting good to excellent reliability.

Compared to the original CDI (Jackson et al., 2022a), the removal of nine items and the reduction to five factors may reflect cultural differences in the conceptualization of cardiac distress, although, without further analysis, it is impossible to rule out the effects of sample representativeness and factorization procedures on determining such differing structures. A heatmap of the items between the two factorial structures is presented in Table S30.

“Role Change and Adaptation to Disease” represents the dominant factor in the questionnaire. The Italian CDI incorporates items from uncertainty, disconnection, depletion, and physical challenges factors into this primary factor, with the “Changes to roles and relationships” factor almost completely incorporated as well (Jackson et al., 2022a). Although this combination could appear as a statistical artifact, the individual items form a coherent pattern, addressing identity transformation (“Thinking I will never be the same again”), functional limitations (“Not being able to return to work or continue working “), physical symptoms (“Having more pain than I can deal with”), and uncertainty (“Being unable to plan for the future “). These elements could represent facets of disease adaptation challenges, and the high internal consistency of the factor suggests that this construct may better capture Italian patients’ adaptation-related distress than separate discrete processes. This interpretation aligns with the literature, which identifies identity-related changes, loss, and adaptation as prevailing themes post-cardiac events (Dreyer et al., 2021; Genardini et al., 2008; Higgins et al., 2007; B. M. Murphy et al., 2022). Nonetheless, further discussion on the potential artifactual nature of this factor is given in the following paragraphs.

The “Social Disconnection” factor comprised mainly disconnection items (e.g., “Being isolated from friends and family”) from the original “Disconnection and Hopelessness” factor (Jackson et al., 2022a), highlighting the potential relevance of this aspect for Italian cardiac patients. This finding aligns with recent research by Sharif-Nia et al. (2025a), who observed that social isolation is associated with cardiac distress through both direct and indirect pathways mediated by loneliness.

The “Cognitive Challenges” factor kept almost the same structure as in the original questionnaire (Jackson et al., 2022a). The “Emotional Overwhelm” factor completely incorporated the “Death Concerns” factor plus other negative symptoms from other factors (e.g., “Being tearful more easily than before”), suggesting an integration of death-related thoughts into a broader psychological response. Finally, the “Health System Challenges” factor replicated the structure from the original questionnaire (Jackson et al., 2022a), except for item 22 (“Having difficulty getting to appointments that I need to attend”), which migrated to the “Cognitive Challenges” factor. Given the vague definition of this item, respondents in the original questionnaire may have interpreted it as referring to healthcare appointments, whereas our sample understood the difficulties as related to memory issues or organizational cognitive load.

While these findings suggest cultural differences in the conceptualization of cardiac distress, limited comparative literature prevents definitive conclusions. Currently, to the best of our knowledge, the only other complete cross-cultural CDI validation is the Turkish version (Afşar et al., 2025). A brief comparison reveals a higher number of dropped items (19) and an equal number of factors (5). However, the five factors in the Turkish CDI retain the same names as the original version and show some relevant differences in the maintained factors compared with the Italian ones (e.g., “Disconnection and hopelessness” was dropped, while “Physical challenges” was maintained).

### 4.3. Bifactor and Higher-Order Representations

Ancillary bifactor indices were computed to evaluate the extent to which a general factor accounted for the common variance among items and whether the retained group factors contributed reliable variance beyond it (Rodriguez et al., 2016). This was particularly informative given the size of the “Role Change and Adaptation to Disease” factor, which comprises 21 items.

Still, omega hierarchical indicated that the general factor accounted for approximately 60% of the variance in total scores, and the explained common variance accounted for roughly one third of the variance shared among items, both below the thresholds conventionally taken to indicate essential unidimensionality. This is consistent with the conceptualization of cardiac distress as a multidimensional construct (Jackson et al., 2022b). Construct replicability was high for the general factor, indicating that it is well defined by the present item set and would be expected to be recovered in other samples.

At the subscale level, omega hierarchical subscale values were consistently low relative to the corresponding subscale omega values, indicating that most of the reliable variance in subscale scores is attributable to the general factor rather than to the specific domain. Each domain retains substantial specific variance, but subscale scores should be read as domain-specific indicators of overall cardiac distress rather than as autonomous measures of their respective domains. Construct replicability exceeded the threshold for all factors except “Social Disconnection”, suggesting replicability in other samples for four factors out of five. The low level of construct replicability for Social Disconnection suggests that this construct may require additional or different items to capture the specificity of this factor in the Italian context. Although these results do not support essential unidimensionality, confirmatory analyses in an independent sample remain necessary before the question can be considered settled.

### 4.4. Criterion Validity and Discriminative Accuracy

The Italian CDI showed good concurrent validity, with moderate to strong correlations with the Distress Thermometer (comparable to, though slightly lower than, the original study), the PHQ-4 and the K6. Discriminant validity showed mixed results: a low correlation with COVID-19 concern and a significant, moderate correlation with anger. The low correlation with COVID-19 Concern supports discriminant validity. On the other hand, the anger correlation, which is similar to the original study (Jackson et al., 2022a), may reflect the role of anger in cardiac distress rather than poor discriminant validity, in line with the literature characterizing anger as part of the emotional response to cardiac events (Higgins et al., 2007). However, this interpretation is post hoc and requires further assessment in an independent sample. Based on the current results, the discriminant validity of the Italian CDI should be viewed as mixed and not definitive.

The discriminative accuracy for identifying distress potentially associated with mental illness was good (sensitivity = 71%, specificity = 84%). The optimal cut-off was ≥ 36 out of a possible 138, corresponding to a standardized value of 26.1 when expressed as a percentage of the maximum possible score. This is similar, although higher, to the standardized value of 22.4 obtained in the original study for severe distress, with comparable sensitivity and specificity (71% and 86%, respectively; Jackson et al., 2022a). Further comparison with other distress criteria (PHQ-4 and Distress Thermometer) presented similar discriminative accuracy, although with quite variable cut-offs. Nonetheless, the K6-based cut-off remains the recommended threshold, as the remaining comparisons were intended solely for sensitivity analyses.

Four subscales demonstrated acceptable discriminative accuracy in identifying elevated distress levels within each domain (sensitivity = 48–93%, specificity = 51–91%), while the Health System Challenges subscale did not demonstrate acceptable accuracy against this criterion, and no cut-off is therefore proposed for that domain. In addition, given the relatively low omega hierarchical coefficients for the subscales and the absence of a specific domain criterion for determining the subscale cut-offs, these should be considered indicative only and always interpreted alongside the total score, rather than as independent measures of the individual domains.

### 4.5. Exploratory Analyses

The exploratory multiple linear regression model showed a very low explained variance (6.2%) for the total CDI score, based on site, cardiac event, and time since the event. This suggests that these variables may have a minimal influence on the score. Given the high heterogeneity of the sample with respect to cardiac events, this initial evaluation may reflect a general distribution of scores across different types of cardiac patients. However, given the nature of this test, it is insufficient to rule out differences in the questionnaire’s factorial structure based on these variables.

Although purely exploratory and with some limitations due to the model’s diagnostics, further assessment of this model’s specific results is warranted. A significant association between the CDI score and the site of administration was identified, with patients recruited in Milano scoring higher than patients recruited in Bologna (*p* = 0.002). This difference may be related to the clinical settings in which the patients were assessed: the early, hospitalized post-surgery setting in Milan and the day-hospital rehabilitation setting in Bologna.

Regarding cardiac events, patients who experienced a myocardial infarction reported significantly higher levels of cardiac distress (*p* = 0.018). However, the particularly wide confidence interval warrants caution in interpreting this result. A similar trend was observed in patients with arrhythmic events, although this result was not significant (*p* = 0.057). In contrast, specific surgical treatments, such as CABG (*p* = 0.020) and heart valve surgery (*p* = 0.054), were associated with lower scores, though the latter association was non-significant. Estimates for unstable angina, aortic surgery, and other conditions were imprecise, with confidence intervals too wide to support any conclusion, reflecting the low frequency of these events, their overlap with other conditions, and the heterogeneity within these categories. Additionally, the time elapsed since the event was not associated with the total CDI score. This aligns with the definition of cardiac distress as a persistent condition (Jackson et al., 2018), but the presence of a skewed distribution towards shorter time windows (Table 2) necessitates additional caution when interpreting this result.

### 4.6. Limitations and Future Directions

The present findings must be interpreted in light of several important limitations.

The face validity sample was older and more predominantly male than the validation sample; comprehension difficulties specific to younger or female patients may therefore have gone undetected during pre-testing.

Content validity analyses revealed that 11 items, the overall scale, and all subscales except the physical limits subscale exhibited poor content validity. Future studies should consider re-evaluating the content validity of the newly proposed solution using an independent panel to determine whether it better reflects cardiac distress in Italy.

Despite standardization efforts, methodological variations across centers cannot be ruled out. The recruitment approach, while employing consecutive sampling at one site, was not designed to ensure population representativeness, thus limiting the generalizability of these findings.

The voluntary participation model may have introduced selection bias. Additionally, self-report measures are susceptible to social desirability bias. Future studies should consider incorporating social desirability checks, objective measures, or informant ratings. The specific clinical composition of the sample, characterized by a significant imbalance in the types of cardiac events, recruitment settings, and the time elapsed since the event, may have resulted in a factorial structure that closely aligns with the prevalent conditions, thereby limiting its generalizability to the broader cardiac population. Although the exploratory analysis of total scores makes this theory less plausible, the evidence remains weak, particularly regarding potential differences in factorial structures across patient types. Therefore, future studies should aim to validate the current structure across multiple samples representing different types of cardiac patients to enhance this tool’s representativeness.

From a psychometric perspective, although the sample size met established EFA guidelines (five participants per item), contemporary recommendations suggest alternative methods for estimating sample size. 

The fifth factor in the retained solution shows some weaknesses. It consists of only four items, shows the weakest loading on the second-order factor (0.273, compared with 0.455–0.654 for the remaining factors), was not suggested by PA in the imputed solution and by PA and MAP in the complete-case analysis, and showed unacceptable discriminative accuracy against the K6 criterion. It is, however, also the factor that reproduces the distinction between interpersonal disconnection and health system challenges, which are separate subscales in the original instrument (Jackson et al., 2022a), which motivated its retention. More generally, since no exploratory method definitively determines the optimal number of factors, and since no confirmatory analysis was conducted in an independent sample, the present results should be regarded as preliminary. Future studies should prioritize confirmatory evaluation of both the four- and five-factor structures.

The violation of proportionality constraints in the Schmid–Leiman transformation suggests that the reported bifactor indices are approximate. Additionally, confirmatory testing of the higher-order model is necessary to obtain definitive results regarding potential unidimensional alternatives.

The substantial divergence from the original eight-factor structure raises questions about cross-cultural validity. Future research should conduct rigorous cross-cultural explorations to determine whether observed differences reflect cultural adaptation or psychometric limitations.

Mixed results regarding discriminant validity warrant further evaluation, particularly regarding the relationship between cardiac distress and anger. Future research should more clearly define the relationship between these two concepts and provide additional measures of discriminant validity.

General and subscale cut-offs were determined using general psychological distress (K6) as a common criterion rather than domain-specific clinical measures, limiting their interpretation as diagnostic thresholds for domain-specific problems. At present, no validated alternative measure of cardiac distress exists in the Italian context, limiting the selection of criteria. Nonetheless, regarding the subscales, future studies employing domain-specific criteria would enable more precise clinical interpretation and targeted intervention planning.

Scale scores were computed as unweighted sums, which corresponds to a constrained measurement model whose assumptions were not formally tested in the present study (McNeish & Wolf, 2020). This is a further reason for the caution expressed above regarding subscale scores and cut-offs. Future confirmatory work should test whether the constraints implied by unweighted sum scoring are tenable, and compare sum scores with optimally weighted alternatives before cut-offs are recommended for clinical use. 

Future research should prioritize confirmatory factor analysis to replicate the five-factor structure, longitudinal validation to assess temporal stability, and expanded cross-cultural investigations. Furthermore, exploring associations with clinical variables (e.g., comorbidity, pharmacotherapy) and sociodemographic factors (e.g., gender, socioeconomic status) could further characterize how cardiac distress manifests. Finally, this study focused on recent cardiac events; future studies should assess construct validity and explore adaptation to chronic cardiovascular conditions, which may entail different psychological needs and coping patterns.

## 5. Conclusions

In light of some reported limitations, the Italian CDI shows promising preliminary psychometric properties. As one of the few Italian tools specifically developed for the comprehensive psychological assessment of cardiac patients, if confirmed through further validation of its structure and cut-offs, its multidimensional structure could allow for specific characterization of patient concerns across distinct domains of disease adaptation, emotional overwhelm, cognitive challenges, social disconnection, and healthcare system challenges. This will potentially enable targeted investigations of specific distress domains in research settings and tailored interventions in clinical practice. In conclusion, the Italian CDI addresses a significant gap in cardiac psychology assessment tools and, after further refinement and validation, it could provide healthcare professionals with a culturally appropriate instrument for a comprehensive psychological evaluation of cardiovascular patients in both clinical and research contexts.

## Figures and Tables

**Table 1:** Content Validity Indices for subscales and κ Coefficients of the CDI items.

N.	Item	κ
**Fear and Uncertainty (CVI/S = 0.71)**		
11	Thinking that I am not the person that I used to be	0.70
28	Thinking I will never be the same again	0.91
32	Thinking my condition might get worse	1.00
**33**	**Avoiding activities that make my heart beat faster**	**0.30**
36	Not knowing what the future holds for me	0.70
**38**	**Being in places and situations that remind me of my heart event**	**0.24**
43	Being unable to plan for the future	0.70
47	Dwelling on my heart condition	0.79
**Disconnection and Hopelessness (CVI/S = 0.72)**		
4	Thinking my friends or family don’t understand how difficult it is living with heart disease	0.81
5	Believing that others don’t have the same confidence in me as they did before my heart problem	0.81
**14**	**Being unable to accept help from others**	**0.24**
25	Being isolated from friends and family	0.91
30	Not being supported by my friends and family in my efforts to manage my heart condition	0.57
31	Feeling lonely	0.81
42	Being disconnected from people in my community	0.57
46	Withdrawing from people	0.41
**Changes to Roles and Relationships (CVI/S = 0.77)**		
3	Being unable to take care of family responsibilities	0.81
9	Not being able to return to work or continue working	1.00
12	Thinking that my heart condition controls my life	1.00
**16**	**Thinking that my family is being overprotective of me**	**0.24**
18	Not being able to go too far from home	0.57
35	Becoming a burden to my family	0.70
39	Having changes in my usual roles	0.70
44	Being concerned about my capacity for sexual activity	0.81
48	Being unavailable to my family and friends	0.79
49	Being too dependent on others	0.66
55	Lacking purpose or meaning in life	0.90
**Overwhelm and Depletion (CVI/S = 0.72)**		
**20**	**Being irritated by little things**	**0.24**
27	Not being able to sustain the lifestyle changes I need to make	0.81
29	Being tearful more easily than before	0.57
34	Being unable to deal with stress	0.91
37	Lacking energy	0.91
45	Avoiding situations and activities	0.57
54	Being emotionally exhausted	0.79
**Cognitive Challenges (CVI/S = 0.44)**		
**21**	**Forgetting things more than before**	**0.24**
23	Having difficulty making decisions	0.57
**26**	**Having difficulty concentrating**	**0.30**
**51**	**Having difficulty remembering things**	**0.21**
Physical Challenges **(CVI/S = 0.81)**		
1	Being physically restricted	1.00
2	Being woken up at night by my racing heart	1.00
7	Having more pain than I can deal with	0.91
19	Having chest discomfort	0.70
24	Being overly aware of my heart in my chest	0.81
40	Not sleeping well	0.70
**50**	**Having bad dreams or nightmares**	**0.25**
52	Being short of breath	0.79
**Health System Challenges (CVI/S = 0.69)**		
6	Not getting clear directions from my health practitioner on how to manage my heart condition	0.91
8	Not having my concerns taken seriously by my health practitioner	0.70
17	Not having access to the health care I need	0.57
**22**	**Having difficulty getting to appointments that I need to attend**	**0.30**
41	Not being able to get as much information as I want about my heart condition	0.70
**Death Concerns (CVI/S = 0.75)**		
10	Thinking about dying	0.81
**13**	**Not knowing how my family will cope if something should happen to me**	**0.34**
15	Being afraid of dying	1.00
53	Not knowing what will happen to other people if I die	0.66

Note. Data were obtained from 11 independent evaluators (4 nurses/cardiology technicians, 4 cardiologists, 3 psychologists). When items lacked ratings, calculations for κ and CVI were adjusted based on the number of available evaluators per item. Bold text indicates items with poor content validity (κ < 0.40) or subscales with insufficient content validity (CVI-S/Ave < 0.79).

**Table 2:** Sociodemographic Characteristics of the Sample.

Characteristic	*n*	%
Gender		
Male	204	69.6
Female	89	30.4
Age groups		
<50	29	9.9
50–59	50	17.1
60–69	103	35.2
70–79	98	33.4
>80	13	4.4
Education		
No formal education	3	1.0
Primary	25	8.5
Secondary	198	67.6
Degree	49	16.7
Post-graduate	18	6.1
Employment status ^a^		
Employee	85	29.1
Self-employed	37	12.7
Unemployed	3	1.0
Retired	157	53.8
Not in the paid workforce ^b^	10	3.4
Current financial strain ^c^		
None	194	66.6
Slight	61	21.0
Moderate	24	8.2
Considerable/extreme	12	4.1
Marital status ^a^		
Never married	26	8.9
Widowed	20	6.8
Divorced or separated	27	9.2
Married or living with partner	219	75.0

Note: ^a^ *n* = 292, as one participant did not answer the question. ^b^ e.g., home duties, students. ^c^ *n* = 291, as two participants did not answer the question.

**Table 3:** Clinical Characteristics of the Sample.

Characteristic	*n*	%
Last acute cardiac events ^a^		
Heart valve surgery	208	71.0
CABG	87	29.7
Aortic artery surgery	33	11.3
Cardiac arrhythmias hospitalization/treatment	33	11.3
AMI	16	5.5
Unstable angina ^b^	13	4.4
PCI	9	3.1
ICD	7	2.4
Aortic dissection	7	2.4
HF	4	1.4
Other		
Other surgical procedures ^c^	24	8.2
Other acute events ^d^	15	5.1
Number of concurrent cardiac events		
1	180	61.4
2	78	26.6
3	18	6.1
≥4	17	5.8
Time since last cardiac event		
Less than 1 week	100	34.1
From 1 week to 1 month	93	31.7
1 to 3 months	82	28.0
More than 3 months	18	6.1
Attended cardiac rehabilitation	148	50.5
Risk Factors		
Dyslipidemia	167	57.0
Hypertension	187	63.8
Obesity	42	14.3
Somatic comorbidities		
Diabetes	54	18.4
Renal disease	29	9.9
Chronic HF	26	8.9
Chronic pulmonary disease	23	7.8
Liver disease	18	6.1
Cancer	16	5.5
Rheumatologic disease	8	2.7
HIV/AIDS	1	0.3
Mental comorbidities		
Anxiety	48	16.4
Depression	14	4.8
History of Anxiety	41	14.0
History of Depression	23	7.8
History of Other Mental Diseases	5	1.7
CCI		
0	177	60.4
1	51	17.4
2	38	13.0
3	14	4.8
≥4	13	4.4

Note. CABG = Coronary Artery Bypass Graft; AMI = Acute Myocardial Infarction; PCI = Percutaneous Coronary Intervention; ICD = Implantable Cardioverter-Defibrillator; HF = Heart Failure; CCI = Charlson Comorbidity Index. ^a^ Reported events correspond to consecutive cardiac events that occurred in the month prior to the most recent cardiac event reported by patients. Given that some patients experienced multiple cardiac events, the percentages do not sum to 100%. ^b^ The reported number refers to cases of unstable angina that resulted in emergency department visits or hospitalization. ^c^ e.g., atrial/ventricular septal defect repair, atrioplasty, left atrial appendage closure. ^d^ e.g., cardiac arrest, cardiogenic shock, pericarditis.

**Table 4:** Item Communalities for the Five-Factor Solution.

Item	Communalities
1	0.329
3	0.356
5	0.617
6	0.727
7	0.376
8	0.590
9	0.402
10	0.563
11	0.539
12	0.551
13	0.562
15	0.576
16	0.376
17	0.588
18	0.257
19	0.374
20	0.427
21	0.832
22	0.385
23	0.651
25	0.720
26	0.641
27	0.382
28	0.553
29	0.463
30	0.561
31	0.657
33	0.471
37	0.426
38	0.628
39	0.481
40	0.391
41	0.664
42	0.720
43	0.458
44	0.487
45	0.530
46	0.801
47	0.549
48	0.489
49	0.316
50	0.458
51	0.776
53	0.646
54	0.622
55	0.856

Note. This table presents results from the first imputed dataset.

**Table 5:** Pattern Matrix of the Five-Factor Solution.

Item		Factor				
N.	Content	1	2	3	4	5
Factor 1: Role Change and Adaptation to Disease						
48	Being unavailable to my family and friends	**0.635**	0.066	0.081	−0.046	0.080
5	Believing that others don’t have the same confidence in me as they did before my heart problem	**0.626**	−0.196	0.211	0.101	0.248
44	Being concerned about my capacity for sexual activity	**0.613**	−0.088	−0.175	0.118	0.257
16	Thinking that my family is being overprotective of me	**0.611**	−0.015	−0.064	0.015	−0.304
11	Thinking that I am not the person that I used to be	**0.602**	0.080	0.232	−0.006	−0.040
28	Thinking I will never be the same again	**0.598**	0.241	0.068	0.000	−0.050
43	Being unable to plan for the future	**0.593**	−0.016	0.082	0.145	0.022
27	Not being able to sustain the lifestyle changes I need to make	**0.565**	0.055	0.095	−0.016	−0.011
49	Being too dependent on others	**0.550**	−0.094	−0.009	0.045	0.104
19	Having chest discomfort	**0.509**	−0.010	0.057	0.054	0.188
38	Being in places and situations that remind me of my heart event	**0.503**	0.136	−0.209	**0.399**	0.167
12	Thinking that my heart condition controls my life	**0.489**	0.129	0.243	0.088	0.127
7	Having more pain than I can deal with	**0.485**	0.191	−0.044	0.072	0.063
45	Avoiding situations and activities	**0.476**	0.283	0.072	0.132	**−0.332**
3	Being unable to take care of family responsibilities	**0.460**	0.146	−0.029	−0.015	0.213
1	Being physically restricted	**0.443**	0.199	0.040	−0.092	0.111
39	Having changes in my usual roles	**0.437**	0.313	0.153	0.030	−0.119
33	Avoiding activities that make my heart beat faster	**0.432**	**0.385**	−0.083	0.108	−0.218
9	Not being able to return to work or continue working	**0.400**	0.254	0.126	−0.127	0.190
18	Not being able to go too far from home	**0.373**	−0.027	0.144	0.125	0.084
37	Lacking energy	**0.368**	0.295	0.259	−0.122	−0.051
Factor 2: Emotional Overwhelm						
53	Not knowing what will happen to other people if I die	0.049	**0.774**	0.082	−0.247	0.033
15	Being afraid of dying	−0.154	**0.740**	−0.033	0.161	0.160
10	Thinking about dying	−0.066	**0.705**	0.101	0.090	0.072
13	Not knowing how my family will cope if something should happen to me	0.178	**0.652**	0.063	−0.307	0.072
29	Being tearful more easily than before	−0.021	**0.648**	0.068	0.078	−0.049
47	Dwelling on my heart condition	0.257	**0.569**	−0.058	0.171	−0.118
54	Being emotionally exhausted	0.140	**0.540**	0.221	0.210	−0.055
50	Having bad dreams or nightmares	0.099	**0.432**	−0.075	0.185	**0.342**
40	Not sleeping well	0.237	**0.411**	0.096	0.108	−0.019
20	Being irritated by little things	0.115	**0.329**	**0.404**	0.041	−0.100
Factor 3: Cognitive challenges						
21	Forgetting things more than before	−0.077	−0.068	**0.948**	−0.019	0.032
51	Having difficulty remembering things	−0.041	0.009	**0.890**	−0.112	0.138
26	Having difficulty concentrating	0.156	0.036	**0.670**	0.174	−0.112
23	Having difficulty making decisions	−0.120	0.180	**0.651**	0.302	−0.142
22	Having difficulty getting to appointments that I need to attend	0.128	0.011	**0.559**	0.036	−0.029
Factor 4: Social Disconnection						
46	Withdrawing from people	**0.325**	0.023	−0.093	**0.797**	−0.206
42	Being disconnected from people in my community	0.180	−0.217	0.253	**0.721**	−0.012
25	Being isolated from friends and family	−0.186	0.226	0.195	**0.709**	0.141
55	Lacking purpose or meaning in life	0.291	−0.123	0.302	**0.582**	0.262
30	Not being supported by my friends and family in my efforts to manage my heart condition	−0.114	0.211	0.035	**0.572**	0.308
31	Feeling lonely	−0.061	0.075	0.224	**0.484**	**0.453**
Factor 5: Health System Challenges						
6	Not getting clear directions from my health practitioner on how to manage my heart condition	0.067	0.131	−0.080	−0.011	**0.824**
41	Not being able to get as much information as I want about my heart condition	0.199	−0.008	0.109	0.135	**0.675**
8	Not having my concerns taken seriously by my health practitioner	0.106	0.195	−0.107	0.231	**0.606**
17	Not having access to the health care I need	**0.407**	−0.145	0.126	−0.067	**0.588**

Note. The present table refers to imputation dataset number 1. Factor loadings of 0.32 or higher are in bold.

**Table 6:** Factor Intercorrelations.

Factor	1	2	3	4	5
1	—				
2	0.384	—			
3	0.293	0.289	—		
4	0.277	0.202	0.242	—	
5	0.195	0.080	0.096	0.216	—

Note. The present table refers to imputation dataset number 1.

**Table 7:** Factor-level Bifactor Indices.

Index	Factor				
	1	2	3	4	5
ω*_S_*	0.942	0.927	0.915	0.932	0.930
ω*_HS_*	0.381	0.408	0.434	0.414	0.349
H	0.817	0.821	0.840	0.760	0.820

Note. The present table refers to imputation dataset number 1. ω*_S_* = omega total for subscale; ω*_HS_* = omega hierarchical for subscale; H = construct replicability index.

**Table 8:** Discriminative Accuracy and Optimal Cut-offs for CDI Subscales.

Subscale	AUC	95% CI	Items	Range	Cut-Off	Sensitivity	Specificity
Role Change and Adaptation to Disease	0.80	0.71–0.86	21	0–63	≥21	61%	86%
Emotional Overwhelm	0.78	0.71–0.84	10	0–30	≥5	93%	51%
Cognitive Challenges	0.73	0.63–0.81	5	0–15	≥1	76%	60%
Social Disconnection	0.72	0.62–0.79	6	0–18	≥2	49%	91%
Health System Challenges ^a^	0.64	0.55–0.71	4	0–12	≥1	42%	84%

Note. Results refer to imputation dataset number 1. AUC = Area Under Curve. ^a^ Given that discriminative accuracy against the K6 was below the conventional threshold of 0.70, this cut-off is reported for completeness but not recommended for use.

**Table 9:** Coefficients of the Exploratory Multiple Linear Regression Model for Total Italian Cardiac Distress Inventory Score.

Predictor	*n*	*B*	95% CI	*p*-Value
Site ^a^	-	7.71	[2.78, 12.63]	0.002
Time from the event	-	0.01	[−0.04, 0.06]	0.777
Heart valve surgery	207	−5.66	[−11.42, 0.09]	0.054
CABG	87	−6.72	[−12.38, −1.05]	0.020
Other events ^b^	53	1.26	[−4.61, 7.12]	0.674
Cardiac arrhythmias hospitalization/treatment	33	6.22	[−0.20, 12.65]	0.057
Aortic Surgery	33	−0.12	[−6.76, 6.51]	0.971
AMI	16	11.34	[1.92, 20.75]	0.018
Unstable angina	13	−6.18	[−16.51, 4.16]	0.240

Note. *n* values are reported only for cardiac events, as these are the only variables coded 0/1. As all patients presented at least one cardiac event, 0 values should be considered as having different cardiac events from the one indicated. ^a^ Reference value for this variable is Bologna. ^b^ This variable includes all cardiac events with a frequency lower than 10 in the sample.

## Data Availability

The data presented in this study are available on request from the corresponding author due to their sensitive nature.

## Supplementary Materials

The following supporting information can be downloaded at: https://www.mdpi.com/article/10.3390/bs16091599/s1, Table S1: Missing Data Rates by Item; Table S2: Kaiser–Meyer–Olkin Sampling Adequacy and Bartlett’s Test of Sphericity Values Across Imputations; Table S3: Eigenvalues of Observed and Simulated Data Throughout the Five Imputations; Table S4: Step-by-step decision regarding item removal in the 5-factor solution; Table S5: Total Variance Explained by Five-Factor Solution Across Imputations; Table S6: Item Communalities for the Five-Factor Solution Across Imputations; Tables S7–S10: Pattern Matrix (Imputation 2–5); Table S11: Factor Intercorrelations Across Imputed Datasets; Table S12: Step-by-step Decision Regarding Item Removal in 4-factor Solution; Table S13: Item Communalities for the Four-Factor Solution; Table S14: Pattern Matrix and Factor Composition for the Four Factor Solution Table S15: Factor Intercorrelations for the Four Factor Solution; Table S16: Item Communalities for the Complete Case Solution; Table S17: Pattern Matrix and Factor Composition for the Complete Case Solution; Table S18: Factor Intercorrelations for the Four Factor Solution; Tables S19–S23: Schmid–Leiman loadings of single items on general and group factors—Imputation 1–5; Table S24: General Factor Bifactor Indices Across Imputations; Table S25: Factor-level Bifactor Indices across imputations; Table S26: Reliability Coefficients Across Imputations for the Five Factor Solution; Table S27: Corrected Item–Total Correlations and Variations in Reliability Coefficients Across Imputations; Table S28: ROC Curve Characteristics for CDI Total Score and Subscales Across Imputations; Table S29: ROC Curve Characteristics for CDI Total Score Across Imputations with PHQ-4 and Distress Thermometer as criteria; Figure S1: ROC Curve comparison of K6, PHQ-4, and Distress Thermometer criteria; Figures S2–S6: ROC Curve for each subscale; Table S30: Item Distribution Across Factors: Comparison of Original and Italian CDI Structure; Italian Cardiac Distress Inventory.

## Abbreviations

The following abbreviations are used in this manuscript:

AMI	Acute myocardial infarction
AUC	Area under the curve
CABG	Coronary artery bypass graft
CCI	Charlson Comorbidity Index
CDI	Cardiac Distress Inventory
CVD	Cardiovascular disease
CVI-I	Content validity index—item
CVI-S/Ave	Content validity index—scale/average
DCPR-SSI	Diagnostic Criteria for Psychosomatic Research Semi-Structured Interview
ECV	Explained Common Variance
EFA	Exploratory factor analysis
ET	Emotion Thermometers
H	Construct replicability
HF	Heart failure
ICD	Implantable cardioverter-defibrillator
ITC	Corrected item–total correlation
K6	Kessler Psychological Distress Scale-6
KMO	Kaiser–Meyer–Olkin
MAR	Missing at random
MAP	Minimum average partial procedure
MCAR	Missing completely at random
MMAS-8	Morisky Medication Adherence Scale-8
PA	Parallel Analysis
PHQ-4	Patient Health Questionnaire-4
PCI	Percutaneous coronary intervention
PSI	PsychoSocial Index
ROC	Receiver operating characteristic
VIF	Variance Inflation Factor
ω*_H_*	Omega hierarchical
ω*_HS_*	Subscale omega hierarchical
ω*_S_*	Subscale omega total
ω*_t_*	Omega total
