# Supplementary material for: Italian Adaptation and Psychometric Validation of the Cardiac Distress Inventory in Patients with Recent Cardiac Events
Source: Behav Sci (Basel). 2026 Sep 8;16(9):1599. doi: 10.3390/bs16091599 (PMC13603514; doi:10.3390/bs16091599)
Supplement: Supplementary file 1 [file behavsci-16-01599-s001.zip › Italian Cardiac Distress Inventory v2.pdf]

Italian Adaptation and Psychometric Validation of the Cardiac Distress Inventory in Patients with Recent Cardiac Events

Graziano Gigante[1], Edward Callus[2,3], Sara Gostoli[1], Enrico Giuseppe Bertoldo[2], Valentina Fiolo[2], Giulia Lorefice[2], Sofia De Stefano[2], Giulia Casu[1], Marco Monti[4], Stefano Urbinati[5], Alun Conrad Jackson[6,7,8], and Chiara Rafanelli[1]

Affiliations:

[1] Department of Psychology "Renzo Canestrari", University of Bologna, Bologna, Italy

[2] Clinical Psychology Service, IRCCS Policlinico San Donato, San Donato Milanese, Italy

[3] Department of Biomedical Sciences for Health, University of Milan, Milan, Italy

[4] Hospital Psychological Service, Bellaria Hospital, AUSL Bologna, Bologna, Italy

[5] Division of Cardiology, Bellaria Hospital, AUSL Bologna, Bologna, Italy

[6] Australian Centre for Heart Health, The Royal Melbourne Hospital, VIC, Australia

[7] School of Psychological Sciences, University of Melbourne, Melbourne, VIC, Australia

[8] Centre on Behavioral Health, University of Hong Kong, Pokfulam, Hong Kong

Corresponding author: Prof. Chiara Rafanelli, PhD, MD: chiara.rafanelli@unibo.it.

Convivere con una malattia cardiaca a volte può essere difficile. Di seguito sono elencate alcune problematiche che le persone che convivono con una malattia cardiaca potrebbero sperimentare. Per favore indichi se ha mai fatto esperienza delle problematiche sotto elencate nel corso delle ultime quattro settimane, segnando - accanto ad ogni problematica - una crocetta su "S" per indicare sì o su "N" per indicare no. Per ogni problematica riscontrata (identificata con una crocetta su "S"), indichi il grado di disagio provocato **nelle ultime quattro settimane** segnando una crocetta su un punteggio su una scala da 0 a 3, in cui 0 indica "nessun disagio" e 3 "disagio grave".

**Problematica**	**Sì**	**No**	**Se sì, per cortesia indichi quanto disagio le ha causato**			
			**Nessun disagio**	**Disagio lieve**	**Disagio moderato**	**Disagio grave**
1. Essere limitato/a fisicamente	S	N	0	1	2	3
2. Non essere in grado di occuparmi delle responsabilità familiari	S	N	0	1	2	3
3. Credere che gli altri non abbiano più la stessa fiducia in me, come avevano prima del mio problema cardiologico	S	N	0	1	2	3
4. Non ricevere dal mio medico indicazioni chiare su come gestire la mia malattia cardiaca	S	N	0	1	2	3
5. Sentire più dolore di quello che posso sopportare	S	N	0	1	2	3
6. Le mie preoccupazioni non sono prese in considerazione seriamente dal mio medico	S	N	0	1	2	3
7. Non essere in grado di tornare al lavoro o di continuare a lavorare	S	N	0	1	2	3
8. Pensare alla morte	S	N	0	1	2	3
9. Pensare di non essere più la persona di un tempo	S	N	0	1	2	3
10. Pensare che la mia malattia cardiaca condizioni la mia vita	S	N	0	1	2	3
11. Non sapere come la mia famiglia affronterà la situazione nel caso in cui dovesse succedermi qualcosa	S	N	0	1	2	3

Problematica	Sì	No	Se sì, per cortesia indichi quanto disagio le ha causato			
			**Nessun disagio**	**Disagio lieve**	**Disagio moderato**	**Disagio grave**
12. Avere paura di morire	S	N	0	1	2	3
13. Pensare che la mia famiglia sia iperprotettiva nei miei confronti	S	N	0	1	2	3
14. Non avere accesso all'assistenza sanitaria di cui ho bisogno	S	N	0	1	2	3
15. Non essere in grado di allontanarmi troppo da casa	S	N	0	1	2	3
16. Avere fastidio al petto	S	N	0	1	2	3
17. Essere irritato/a da piccole cose	S	N	0	1	2	3
18. Dimenticare le cose più spesso di prima	S	N	0	1	2	3
19. Avere difficoltà a recarmi ad appuntamenti già fissati	S	N	0	1	2	3
20. Avere difficoltà nel prendere decisioni	S	N	0	1	2	3
21. Essere isolato/a da amici e familiari	S	N	0	1	2	3
22. Avere difficoltà di concentrazione	S	N	0	1	2	3
23. Non essere in grado di modificare il mio stile di vita, come sarebbe necessario	S	N	0	1	2	3
24. Pensare che non sarò mai più la stessa persona	S	N	0	1	2	3
25. Piangere più facilmente di prima	S	N	0	1	2	3
26. Non sentirmi sostenuto/a da amici e familiari nello sforzo di gestire la mia situazione cardiaca	S	N	0	1	2	3
27. Sentirmi solo/a	S	N	0	1	2	3

Problematica	Sì	No	Se sì, per cortesia indichi quanto disagio le ha causato			
			Nessun disagio	Disagio lieve	Disagio moderato	Disagio grave
28. Evitare attività che aumentano il battito cardiaco	S	N	0	1	2	3
29. Mancanza di energia	S	N	0	1	2	3
30. Trovarmi in luoghi e situazioni che mi ricordano il mio attacco cardiaco	S	N	0	1	2	3
31. Dover modificare i miei ruoli abituali	S	N	0	1	2	3
32. Non dormire bene	S	N	0	1	2	3
33. Non essere in grado di ricevere tutte le informazioni di cui ho bisogno sulla mia malattia cardiaca	S	N	0	1	2	3
34. Essere distaccato dai miei amici e dai miei conoscenti	S	N	0	1	2	3
35. Non essere in grado di pianificare il futuro	S	N	0	1	2	3
36. Avere preoccupazioni relative alla mia attività sessuale	S	N	0	1	2	3
37. Evitare situazioni e attività	S	N	0	1	2	3
38. Allontanarmi dalle persone	S	N	0	1	2	3
39. Pensare costantemente alla mia situazione cardiaca	S	N	0	1	2	3
40. Non essere disponibile per la mia famiglia e i miei amici	S	N	0	1	2	3
41. Essere troppo dipendente dagli altri	S	N	0	1	2	3
42. Fare brutti sogni o avere incubi	S	N	0	1	2	3
43. Avere difficoltà nel ricordare le cose	S	N	0	1	2	3

Problematica	Sì	No	Se sì, per cortesia indichi quanto disagio le ha causato			
			**Nessun disagio**	**Disagio lieve**	**Disagio moderato**	**Disagio grave**
44. Non sapere cosa potrebbe accadere alle altre persone nel caso in cui morissi	S	N	0	1	2	3
45. Essere emotivamente provato/a	S	N	0	1	2	3
46. Mancanza di scopo o significato nella vita	S	N	0	1	2	3

SCORING DELLA TRADUZIONE ITALIANA DEL CARDIAC DISTRESS INVENTORY

Il Cardiac Distress Inventory è uno strumento composto da 46 item suddivisi in 5 sottoscale: Cambiamento di Ruolo e Adattamento alla Malattia (21 item); Sovraccarico Emotivo (10 item); Sfide Cognitive (5 item); Disconnessione Sociale (6 item); Sfide Riguardanti il Sistema Sanitario (4 item).

Tutti gli item sono valutati da 0 a 3:
0 - item non presente OPPURE item presente ma nessun disagio associato
1 - item presente con associato un basso livello di disagio
2 - item presente con associato un livello di disagio medio
3 - item presente con associato un livello di disagio alto

Cambiamento di Ruolo e Adattamento alla Malattia - 21 item

Somma degli item: 01, 02, 03, 05, 07, 09, 10, 13, 15, 16, 23, 24, 28, 29, 30, 31, 35, 36, 37, 40, 41. Un punteggio pari o superiore a 21 è indicatore di distress severo.
È possibile ottenere un punteggio standardizzato in centesimi dividendo la somma degli item/il massimo punteggio totale possibile (63) x 100

Sovraccarico Emotivo - 10 item

Somma degli item: 08, 11, 12, 17, 25, 32, 39, 42, 44, 45. Un punteggio pari o superiore a 5 è indicatore di distress severo.
È possibile ottenere un punteggio standardizzato in centesimi dividendo la somma degli item/il massimo punteggio totale possibile (30) x 100

Sfide Cognitive - 5 item

Somma degli item: 18, 19, 20, 22, 43. Un punteggio pari o superiore a 1 è indicatore di distress severo.
È possibile ottenere un punteggio standardizzato in centesimi dividendo la somma degli item/il massimo punteggio totale possibile (15) x 100

Disconnessione Sociale - 6 item

Somma degli item: 21, 26, 27, 34, 38, 46. Un punteggio pari o superiore a 2 è indicatore di distress severo.
È possibile ottenere un punteggio standardizzato in centesimi dividendo la somma degli item/il massimo punteggio totale possibile (18) x 100

Sfide Riguardanti il Sistema Sanitario - 4 item

Somma degli item: 04, 06, 14, 33. Un punteggio pari o superiore a 1 è indicatore di distress severo.
È possibile ottenere un punteggio standardizzato in centesimi dividendo la somma degli item/il massimo punteggio totale possibile (12) x 100

Nota. Il cut-off della sottoscala Sfide Riguardanti il Sistema Sanitario ha mostrato un'accuratezza discriminativa inferiore alla soglia convenzionale di .70 (AUC = .635) rispetto al criterio esterno adottato. È riportato per completezza, ma non se ne raccomanda l'uso come indicatore autonomo.

PUNTEGGIO TOTALE CDI - 46 item

Somma di tutti gli item. Un punteggio pari o superiore a 36 è indicatore di distress severo.
È possibile ottenere un punteggio standardizzato in centesimi dividendo la somma degli item/il massimo punteggio totale possibile (138) x 100.

Per gli item mancanti sostituire con il valore medio degli item presenti, purché almeno metà delle risposte sia stata compilata (23 item).
