# Supplementary material for: Italian Adaptation and Psychometric Validation of the Cardiac Distress Inventory in Patients with Recent Cardiac Events
Source: Behav Sci (Basel). 2026 Sep 8;16(9):1599. doi: 10.3390/bs16091599 (PMC13603514; doi:10.3390/bs16091599)
Supplement: Supplementary file 1 [file behavsci-16-01599-s001.zip › Supplementary Materials.pdf]

Table S1

Missing Data Rates by Item

Item	Complete	Missing	
	n	*n*	%
Italian Cardiac Distress Inventory			
CDI 1	290	3	1.0
CDI 2	293	0	0.0
CDI 3	291	2	0.7
CDI 4	293	0	0.0
CDI 5	293	0	0.0
CDI 6	291	2	0.7
CDI 7	293	0	0.0
CDI 8	293	0	0.0
CDI 9	293	0	0.0
CDI 10	290	3	1.0
CDI 11	291	2	0.7
CDI 12	292	1	0.3
CDI 13	292	1	0.3
CDI 14	293	0	0.0
CDI 15	292	1	0.3
CDI 16	291	2	0.7
CDI 17	292	1	0.3
CDI 18	292	1	0.3
CDI 19	291	2	0.7
CDI 20	293	0	0.0
CDI 21	293	0	0.0
CDI 22	293	0	0.0
CDI 23	293	0	0.0

Item	Complete	Missing	
	n	*n*	%
CDI 24	290	3	1.0
CDI 25	293	0	0.0
CDI 26	293	0	0.0
CDI 27	291	2	0.7
CDI 28	293	0	0.0
CDI 29	293	0	0.0
CDI 30	293	0	0.0
CDI 31	293	0	0.0
CDI 32	293	0	0.0
CDI 33	290	3	1.0
CDI 34	292	1	0.3
CDI 35	293	0	0.0
CDI 36	291	2	0.7
CDI 37	292	1	0.3
CDI 38	293	0	0.0
CDI 39	292	1	0.3
CDI 40	290	3	1.0
CDI 41	291	2	0.7
CDI 42	293	0	0.0
CDI 43	292	1	0.3
CDI 44	291	2	0.7
CDI 45	292	1	0.3
CDI 46	293	0	0.0
CDI 47	293	0	0.0
CDI 48	293	0	0.0
CDI 49	293	0	0.0

Item	Complete	Missing	
	n	*n*	%
CDI 50	293	0	0.0
CDI 51	292	1	0.3
CDI 52	290	3	1.0
CDI 53	292	1	0.3
CDI 54	293	0	0.0
CDI 55	293	0	0.0
Emotion Thermometers + COVID-19 Concern			
Distress ET	291	2	0.7
Anxiety ET	291	2	0.7
Depression ET	291	2	0.7
Anger ET	291	2	0.7
COVID-19 Concern	290	3	1.0
Kessler-6 Psychological Distress Scale			
K6 D1 a	292	1	0.3
K6 D1 b	292	1	0.3
K6 D1 c	292	1	0.3
K6 D1 d	293	0	0.0
K6 D1 e	293	0	0.0
K6 D1 f	293	0	0.0

Note. CDI and K6 items are labeled with scale name and item number or identifier. CDI = Cardiac Distress Inventory; ET = Emotion Thermometers; K6 = Kessler-6 Psychological Distress Scale.

Exploratory Factor Analysis

Complete-case analysis statistics are provided in Tables S2-S3, in the section "Complete Case Exploratory Factor Analysis", and in Tables S16-S18.

Table S2

Kaiser-Meyer-Olkin Sampling Adequacy and Bartlett's Test of Sphericity Values Across Imputations

Imputation number	KMO	Bartlett's test of sphericity		
		χ^2	*df*	*p*
Original Data	.64	62,023.6	1485	< .001***
1	.65	65,399.3	1485	< .001***
2	.65	65,460.8	1485	< .001***
3	.65	65,368.5	1485	< .001***
4	.65	65,473.0	1485	< .001***
5	.65	65,326.5	1485	< .001***

Note. Original data: n = 277; imputed data: N = 293. KMO = Kaiser-Meyer-Olkin measure of sampling adequacy.

*** p < .001.

Table S3

Eigenvalues of Observed and Simulated Data Throughout the Five Imputations

Factor	Observed	Simulated	Observed	Simulated	Observed	Simulated	Observed	Simulated	Observed	Simulated	Observed	Simulated
	Original data		Imputation 1		Imputation 2		Imputation 3		Imputation 4		Imputation 5	
1	17.07	3.64	17.10	3.67	17.10	3.63	17.12	3.60	17.12	3.63	17.07	3.59
2	3.76	3.05	3.55	3.05	3.58	3.02	3.54	3.03	3.58	2.63	3.55	3.00
3	3.13	2.63	2.97	2.61	3.01	2.58	2.96	2.62	2.99	2.60	2.98	2.62
**4**	**2.52**	**2.36**	**2.37**	**2.35**	**2.39**	**2.30**	**2.35**	**2.33**	**2.38**	**2.32**	**2.36**	**2.33**
5	1.98	2.13	1.99	2.14	2.02	2.11	1.97	2.13	2.01	2.15	1.95	2.11
6	1.84	2.00	1.76	2.01	1.77	1.96	1.76	2.00	1.78	2.00	1.75	2.00
7	1.56	1.87	1.54	1.88	1.55	1.84	1.52	1.87	1.55	1.87	1.50	1.87
8	1.36	1.76	1.31	1.77	1.31	1.71	1.30	1.76	1.33	1.77	1.30	1.76
9	1.19	1.64	1.18	1.65	1.21	1.63	1.17	1.67	1.20	1.66	1.16	1.65
10	1.13	1.54	1.04	1.55	1.07	1.53	1.04	1.58	1.07	1.57	1.04	1.56
11	1.08	1.49	1.01	1.50	1.02	1.45	-	-	1.03	1.47	-	-
12	1.01	1.40	-	-	-	-	-	-	-	-	-	-

Note. Data are reported for all observed eigenvalues greater than 1, in accordance with the Kaiser criterion. Simulated values correspond to the 95th percentile of the permuted distribution. Bold values indicate the last factor for which the observed eigenvalue exceeds the corresponding simulated value.

Table S4

Step-by-step decision regarding item removal in the 5-factor solution

Step	Item(s)	Decision
1	4, 14, 24, 32, 34, 35, 36	**Removed due to loadings under threshold** (.272 - .312). EFA was fitted again.
2	52	**Removed due to loadings under threshold** (.319). EFA was fitted again. No further items with loading under the threshold were identified.
3	33 ("Avoiding activities that make my heart beat faster")	**Cross-loading on factors one (.418) and two (.376)**. **The item was retained on factor** one because it was theoretically more closely associated with adaptation to disease than with emotional overwhelm.
4	50 ("Having bad dreams or nightmares")	**Cross-loading on factors two (.415) and five (.362)**. **The item was retained on factor two** because it was theoretically more closely associated with emotional overwhelm than with health system challenges.
5	38 ("Being in places and situations that remind me of my heart event")	**Cross-loading on factors one (.489) and four (.419). The item was retained on factor one** because it was theoretically more closely associated with adaptation to disease than with social disconnection.
6	20 ("Being irritated by little things")	**Cross-loading on factors three (.410) and two (.330)**. Although irritability could be associated with cognitive difficulties, the strongest theoretical association was clearly with factor two, emotional overwhelm. Therefore, **the item was reassigned to factor two.**

7	30 ("Not being supported by my friends and family in my efforts to manage my heart condition")	**Low ITC on factor four (.201)**, **associated with a non-significant decrease in reliability after removal (- .003). The item was maintained** due to its theoretical relevance and added value in this factor.
8	38 ("Being in places and situations that remind me of my heart event")	**Low ITC on factor one (.206), associated with a non-significant decrease in reliability after removal (- .004). The item was maintained** due to its theoretical relevance and added value in this factor.
9	2 ("Being woken up at night by my racing heart")	**Low ITC on factor four (.225), associated with an increase in reliability after removal (.018). The item was removed** due to a non-relevant theoretical association with this factor. EFA was fitted again. No further items with loading under the threshold were identified.
10	31 ("Feeling lonely")	**Cross-loading on factors four (.481) and five (.458). The item was retained on factor four** because it was theoretically more closely associated with social disconnection than with health system challenges. No further cross-loadings were identified.
11	44 ("Being concerned about my capacity for sexual activity")	**Low ITC on factor one (.241), associated with a non-significant decrease in reliability after removal (- .002). The item was maintained** due to its theoretical relevance and added value in this factor.
12	16 ("Thinking that my heart condition controls my life")	**Low ITC on factor one (.262), associated with a non-significant decrease in reliability after removal (- .001). The item was maintained** due to its theoretical relevance and added value in this factor.

13	5 ("Believing that others don't have the same confidence in me as they did before my heart problem")	**Low ITC on factor one (.290), associated with a non-significant decrease in reliability after removal (- .004). The item was maintained** due to its theoretical relevance and added value in this factor. No further ITC items were identified.

Note. The present table refers to imputation 1, as potential differences between imputations were treated conservatively and reported, and final decisions were equal across imputations. The name of the item is reported in cases where theoretical evaluations were conducted. Causes of evaluation of the items and the respective decisions are presented in bold. EFA = Exploratory Factor Analysis; ITC = Item-Total Correlation.

Table S5

Total Variance Explained by Five-Factor Solution Across Imputations

Imputation number	Explained variance
1	54.0%
2	53.9%
3	54.1%
4	54.0%
5	53.9%

Table S6

Item Communalities for the Five-Factor Solution Across Imputations

Item	Imputation				
	1	2	3	4	5
1	.329	.321	.330	.331	.322
3	.356	.353	.356	.352	.330
5	.617	.624	.617	.616	.625
6	.727	.705	.725	.724	.726
7	.376	.376	.376	.376	.374
8	.590	.590	.588	.590	.586
9	.402	.398	.402	.401	.402
10	.563	.577	.576	.573	.569
11	.539	.540	.541	.536	.540
12	.551	.551	.552	.550	.551
13	.562	.561	.558	.562	.560
15	.576	.584	.587	.577	.587
16	.376	.371	.378	.379	.380
17	.588	.582	.586	.587	.585
18	.257	.256	.255	.257	.254

Item	Imputation				
	1	2	3	4	5
19	.374	.376	.379	.376	.378
20	.427	.425	.427	.427	.427
21	.832	.831	.830	.831	.825
22	.385	.382	.387	.385	.391
23	.651	.648	.651	.652	.651
25	.720	.720	.719	.721	.721
26	.641	.640	.640	.640	.640
27	.382	.380	.382	.383	.381
28	.553	.552	.554	.552	.553
29	.463	.460	.462	.464	.466
30	.561	.564	.557	.563	.555
31	.657	.656	.664	.658	.663
33	.471	.473	.473	.465	.460
37	.426	.424	.426	.427	.427
38	.628	.628	.628	.628	.630
39	.481	.486	.481	.482	.482
40	.391	.397	.396	.395	.396
41	.664	.659	.665	.666	.663
42	.720	.722	.723	.721	.720
43	.458	.455	.459	.460	.459
44	.487	.484	.492	.489	.492
45	.530	.537	.530	.529	.527
46	.801	.808	.802	.800	.812
47	.549	.548	.551	.550	.550
48	.489	.489	.489	.492	.488
49	.316	.323	.311	.321	.321

Item	Imputation				
	1	2	3	4	5
50	.458	.456	.453	.451	.450
51	.776	.774	.775	.776	.758
53	.646	.641	.654	.648	.653
54	.622	.622	.622	.623	.623
55	.856	.855	.856	.856	.857

Note. The present table refers to the final 46-item solution.

Pattern Matrix for Imputation 1 is reported in the main text, in Table 5.

Table S7

Pattern Matrix (Imputation 2)

Item	Factor				
	1	2	3	4	5
48	**.633**	.066	.082	-.042	.079
5	**.632**	-.202	.215	.104	.239
44	**.609**	-.090	-.175	.125	.253
16	**.609**	-.013	-.062	.015	-.297
11	**.610**	.066	.232	.001	-.048
28	**.599**	.237	.070	.004	-.048
43	**.586**	-.011	.081	.146	.029
27	**.564**	.053	.095	-.008	-.019
49	**.552**	-.094	-.009	.039	.116
19	**.505**	-.007	.055	.045	.204
38	**.500**	.134	-.209	**.403**	.168
12	**.491**	.121	.242	.092	.129

Item	Factor				
	1	2	3	4	5
7	**.479**	.195	-.045	.067	.082
45	**.480**	.279	.073	.141	**-.342**
3	**.450**	.154	-.034	-.017	.219
1	**.441**	.193	.035	-.094	.114
39	**.445**	.306	.155	.036	-.129
33	**.434**	**.382**	-.081	.115	-.224
9	**.400**	.252	.127	-.121	.184
18	**.369**	-.025	.142	.124	.094
37	**.367**	.294	.259	-.123	-.041
53	.052	**.773**	.076	-.245	.026
15	-.159	**.745**	-.034	.159	.166
10	-.072	**.713**	.113	.090	.063
13	.178	**.653**	.063	-.301	.066
29	-.014	**.642**	.069	.081	-.054
47	.257	**.567**	-.058	.173	-.117
54	.143	**.537**	.221	.210	-.052
50	.101	**.428**	-.073	.185	**.341**
40	.246	**.406**	.103	.105	-.011
21	-.074	-.071	**.948**	-.015	.019
51	-.040	.008	**.889**	-.112	.135
26	.154	.035	**.672**	.173	-.107
23	-.121	.181	**.651**	.298	-.135
22	.123	.014	**.558**	.029	-.010
20	.115	**.329**	**.405**	.037	-.091
46	**.323**	.019	-.093	**.805**	-.210
42	.176	-.221	.254	**.724**	-.016

Item	Factor				
	1	2	3	4	5
25	-.189	.224	.194	**.710**	.140
55	.286	-.125	.302	**.585**	.259
30	-.120	.212	.033	**.572**	.312
31	-.065	.074	.222	**.483**	**.455**
6	.074	.117	-.057	-.018	**.812**
41	.194	-.008	.107	.137	**.673**
8	.100	.195	-.108	.232	**.608**
17	**.399**	-.141	.122	-.063	**.586**

Note. The present table refers to the final 46-item solution. Factor loadings ≥ .32 are in bold.

Table S8

Pattern Matrix (Imputation 3)

Item	Factor				
	1	2	3	4	5
48	**.632**	.064	.083	-.045	.085
5	**.621**	-.200	.214	.099	.253
44	**.616**	-.105	-.172	.117	.261
16	**.617**	-.018	-.062	.018	-.302
11	**.605**	.076	.233	-.005	-.037
28	**.602**	.236	.069	.000	-.045
43	**.592**	-.018	.084	.145	.025
27	**.567**	.052	.096	-.016	-.007
49	**.539**	-.084	-.009	.043	.111
19	**.504**	-.002	.059	.053	.195
38	**.500**	.133	-.210	**.397**	.178

Item	Factor				
	1	2	3	4	5
12	**.489**	.125	.244	.088	.133
7	**.478**	.198	-.047	.074	.071
45	**.485**	.281	.071	.134	**-.328**
3	**.455**	.145	-.030	-.015	.219
1	**.443**	.196	.041	-.091	.115
39	**.442**	.311	.151	.029	-.112
33	**.437**	**.386**	-.086	.109	-.211
9	**.401**	.248	.126	-.129	.194
18	**.365**	-.020	.142	.127	.090
37	**.369**	.297	.256	-.122	-.045
53	.047	**.781**	.075	-.248	.040
15	-.162	**.741**	-.024	.177	.154
10	-.063	**.706**	.121	.091	.055
13	.175	**.651**	.056	-.308	.078
29	-.016	**.648**	.063	.076	-.042
47	.259	**.571**	-.063	.172	-.110
54	.144	**.541**	.217	.209	-.048
50	.101	**.420**	-.075	.182	**.349**
40	.248	**.407**	.099	.105	-.011
21	-.077	-.064	**.947**	-.019	.028
51	-.042	.011	**.888**	-.113	.135
26	.159	.038	**.669**	.173	-.110
23	-.117	.185	**.649**	.301	-.141
22	.131	.009	**.561**	.035	-.030
20	.116	**.335**	**.400**	.041	-.096
46	**.325**	.029	-.095	**.799**	-.197

Item	Factor				
	1	2	3	4	5
42	.181	-.221	.256	**.721**	-.010
25	-.185	.223	.193	**.707**	.146
55	.289	-.130	.305	**.578**	.266
30	-.114	.206	.036	**.565**	.313
31	-.074	.083	.221	**.481**	**.463**
6	.059	.118	-.080	-.015	**.829**
41	.188	-.012	.109	.130	**.683**
8	.098	.188	-.106	.228	**.611**
17	**.393**	-.146	.127	-.069	**.593**

Note. The present table refers to the final 46-item solution. Factor loadings ≥ .32 are in bold.

Table S9

Pattern Matrix (Imputation 4)

Item	Factor				
	1	2	3	4	5
48	**.635**	.067	.083	-.046	.082
5	**.622**	-.194	.213	.100	.251
44	**.616**	-.098	-.172	.118	.258
16	**.614**	-.014	-.062	.016	-.304
11	**.596**	.085	.233	-.004	-.037
28	**.593**	.245	.069	.002	-.046
43	**.592**	-.014	.083	.146	.023
27	**.568**	.053	.094	-.015	-.016
49	**.553**	-.094	-.007	.042	.107
19	**.507**	-.004	.058	.050	.193

Item	Factor				
	1	2	3	4	5
38	**.500**	.136	-.208	**.398**	.174
12	**.482**	.133	.243	.089	.131
7	**.481**	.196	-.045	.071	.068
45	**.474**	.288	.070	.135	**-.328**
3	**.455**	.150	-.034	-.015	.212
1	**.443**	.202	.040	-.094	.114
39	**.436**	.317	.152	.030	-.114
33	**.427**	**.386**	-.087	.112	-.216
9	**.393**	.258	.125	-.125	.193
18	**.378**	-.029	.142	.124	.081
37	**.366**	.299	.258	-.122	-.047
53	.046	**.777**	.079	-.246	.038
15	-.158	**.739**	-.034	.163	.162
10	-.064	**.705**	.115	.095	.057
13	.172	**.655**	.062	-.304	.075
29	-.021	**.649**	.066	.078	-.043
47	.253	**.572**	-.060	.172	-.112
54	.138	**.543**	.219	.210	-.049
50	.101	**.420**	-.073	.184	**.346**
40	.243	**.407**	.102	.105	-.013
21	-.077	-.067	**.948**	-.020	.031
51	-.042	.009	**.889**	-.113	.137
26	.155	.037	**.670**	.174	-.111
23	-.117	.179	**.651**	.303	-.142
22	.127	.013	**.559**	.036	-.029
20	.116	**.330**	**.404**	.040	-.098

Item	Factor				
	1	2	3	4	5
46	**.327**	.022	-.092	**.797**	-.202
42	.180	-.218	.253	**.721**	-.010
25	-.188	.225	.193	**.709**	.146
55	.289	-.126	.303	**.581**	.265
30	-.117	.209	.035	**.571**	.312
31	-.064	.072	.225	**.480**	**.458**
6	.064	.122	-.080	-.012	**.825**
41	.193	-.009	.110	.132	**.680**
8	.099	.193	-.107	.231	**.609**
17	**.401**	-.144	.127	-.069	**.590**

Note. The present table refers to the final 46-item solution. Factor loadings ≥ .32 are in bold.

Table S10

Pattern Matrix (Imputation 5)

Item	Factor				
	1	2	3	4	5
48	**.632**	.068	.078	-.040	.080
5	**.630**	-.201	.217	.098	.249
44	**.618**	-.101	-.176	.124	.254
16	**.614**	-.012	-.063	.026	-.310
11	**.602**	.074	.238	-.007	-.036
28	**.597**	.241	.070	.003	-.048
43	**.590**	-.017	.089	.144	.024
27	**.562**	.056	.098	-.014	-.009
49	**.552**	-.084	-.013	.043	.108

Item	Factor				
	1	2	3	4	5
19	**.502**	.002	.064	.051	.194
38	**.501**	.137	-.208	**.400**	.173
12	**.488**	.122	.246	.086	.134
7	**.473**	.203	-.042	.071	.072
45	**.477**	.287	.076	.133	**-.326**
3	**.440**	.145	-.041	-.022	.211
1	**.435**	.198	.037	-.099	.119
39	**.439**	.316	.150	.029	-.113
33	**.419**	**.389**	-.063	.106	-.213
9	**.395**	.251	.130	-.133	.197
18	**.362**	-.016	.147	.122	.093
37	**.361**	.301	.260	-.127	-.042
53	.046	**.780**	.072	-.251	.042
15	-.169	**.742**	-.021	.175	.156
10	-.062	**.703**	.113	.094	.057
13	.171	**.652**	.059	-.305	.076
29	-.020	**.655**	.057	.076	-.040
47	.251	**.576**	-.057	.170	-.108
54	.140	**.545**	.217	.206	-.046
50	.104	**.418**	-.078	.183	**.345**
40	.244	**.408**	.101	.104	-.011
21	-.078	-.067	**.945**	-.024	.032
51	-.047	.007	**.884**	-.101	.113
26	.155	.038	**.673**	.168	-.107
23	-.120	.184	**.655**	.291	-.133
22	.126	.007	**.569**	.026	-.024

Item	Factor				
	1	2	3	4	5
20	.112	**.337**	**.400**	.039	-.095
46	**.323**	.037	-.095	**.806**	-.200
42	.180	-.217	.260	**.716**	-.005
25	-.188	.226	.197	**.702**	.152
55	.291	-.131	.312	**.573**	.269
30	-.114	.206	.042	**.558**	**.320**
31	-.072	.082	.222	**.475**	**.468**
6	.061	.116	-.083	-.017	**.829**
41	.192	-.013	.104	.129	**.682**
8	.098	.187	-.099	.223	**.612**
17	**.398**	-.146	.123	-.070	**.591**

Note. The present table refers to the final 46-item solution. Factor loadings ≥ .32 are in bold.

Table S11

Factor Intercorrelations Across Imputed Datasets

Factor	1	2	3	4	5
			Imputation 1		
1	—				
2	.384	—			
3	.293	.289	—		
4	.277	.202	.242	—	
5	.195	.080	.096	.216	—
			Imputation 2		
1	—				
2	.382	—			

Factor	1	2	3	4	5
3	.291	.289	—		
4	.277	204	.245	—	
5	.199	.080	.102	.219	—
			Imputation 3		
1	—				
2	.382	—			
3	.291	.290	—		
4	.273	202	.243	—	
5	.206	.086	.105	.220	—
			Imputation 4		
1	—				
2	.383	—			
3	.291	.289	—		
4	.274	203	.243	—	
5	.199	.084	.100	.217	—
			Imputation 5		
1	—				
2	.384	—			
3	.291	.296	—		
4	.267	200	.244	—	
5	.206	.090	.104	.219	—

4-Factors Solution Exploratory Factor Analysis

Given the stability of the number of retained factors (equivalence across imputations), the close similarity of loadings (maximum absolute difference = .035; Tucker's congruence coefficients = 1.00), the convergence of item removal decisions across imputed datasets, and the sensitivity nature of this analysis, only the results from the first dataset are reported.

Table S12

Step-by-step Decision Regarding Item Removal in 4-factor Solution

Step	Item(s)	Decision
1	2, 14, 35, 36, 46	**Items 2, 14, and 35 were removed due to loadings under threshold (.266 - .315). Items 36 and 46 were below the threshold in two (.313-314) and four (.313-320[a]) out of five imputations, but were retained** due to the declared conservative approach. EFA was fitted again.
2	24, 34, 36, 46, 32	**Items 24, 34, 36, and 46 were removed due to loadings under threshold (.302 - .313). Item 32 was below the threshold in four out of five imputations (.312-.315), but was retained** due to the declared conservative approach. EFA was fitted again.
3	52	**Was below the threshold in four out of five imputations (.313-.320[a]), but was retained** due to the declared conservative approach.

4	33 ("Avoiding activities that make my heart beat faster")	**Cross-loading on factors one (.412) and two (.399)**. **The item was retained on factor** one because it was theoretically more closely associated with adaptation to disease than with emotional overwhelm.
5	50 ("Having bad dreams or nightmares")	**Cross-loading on factors four (.426) and two (.398)**. **The item was reassigned to factor two** because it was theoretically more closely associated with emotional overwhelm than with social support.
6	17 ("Not having access to the health care I need")	**Cross-loading on factors one (.473) and four (.414). The item was reassigned to factor four** because it was theoretically more closely associated with social support than with adaptation to disease.
7	55 ("Lacking purpose or meaning in life")	**Cross-loading on factors four (.541) and three (.466). The item was removed** due to a non-relevant theoretical association with these factors. EFA was fitted again. No further items with loading under the threshold or cross-loadings were identified.
8	38 ("Being in places and situations that remind me of my heart event")	**Low ITC on factor one (.204), associated with a non-significant decrease in reliability after removal (- .003). The item was maintained** due to its theoretical relevance and added value in this factor.
9	42 ("Being disconnected from people in my community")	**Low ITC on factor three (.213)**, **associated with an increase in reliability after removal (.005). The item was removed** due to a non-relevant theoretical association with this factor. EFA was fitted again. No further items with loading under the threshold were identified.

10	44 ("Being concerned about my capacity for sexual activity")	**Low ITC on factor one (.239), associated with a non-significant decrease in reliability after removal (- .003). The item was maintained** due to its theoretical relevance and added value in this factor.
11	4 ("Thinking my friends or family don't understand how difficult it is living with heart disease ")	**Low ITC on factor four (.239), associated with a non-significant decrease in reliability after removal (- .003). The item was maintained** due to its theoretical relevance and added value in this factor.
12	25 ("Being isolated from friends and family")	**Low ITC on factor four (.250), associated with a non-significant decrease in reliability after removal (- .009). The item was maintained** due to its theoretical relevance and added value in this factor.
13	16 ("Thinking that my family is being overprotective of me")	**Low ITC on factor one (.265), associated with a non-significant decrease in reliability after removal (- .001). The item was maintained** due to its theoretical relevance and added value in this factor.
14	5 ("Believing that others don't have the same confidence in me as they did before my heart problem")	**Low ITC on factor one (.298), associated with a non-significant decrease in reliability after removal (- .005). The item was maintained** due to its theoretical relevance and added value in this factor. No further ITCs were identified.

Note. The present table refers to imputation 1, as potential differences between imputations were treated conservatively and reported, and final decisions were therefore equal across imputations. The name of the item is reported in cases where theoretical evaluations were conducted. Causes

of evaluation of the items and the respective decisions are presented in bold. EFA = Exploratory Factor Analysis; ITC = Item-Total Correlation.

a. The reported number appears to be over the threshold due to an approximation to the 3th decimal place.

Table S13

Item Communalities for the Four-Factor Solution

Item	Communalities
1	.329
3	.356
5	.617
6	.727
7	.376
8	.590
9	.402
10	.563
11	.539
12	.551
13	.562
15	.576
16	.376
17	.588
18	.257
19	.374
20	.427
21	.832
22	.385
23	.651
25	.720
26	.641
27	.382
28	.553
29	.463

Item	Communalities
30	.561
31	.657
33	.471
37	.426
38	.628
39	.481
40	.391
41	.664
42	.720
43	.458
44	.487
45	.530
46	.801
47	.549
48	.489
49	.316
50	.458
51	.776
53	.646
54	.622
55	.856

Note. This table presents results from the first imputed dataset and refers to the final 46-item solution.

Table S14

Pattern Matrix and Factor Composition for the Four Factor Solution

Item	Factor			
	1	2	3	4
	Factor 1			
5	**.669**	-.199	.184	.206
48	**.662**	.047	.045	-.006
44	**.649**	-.094	-.168	.239
11	**.609**	.074	.237	-.075
28	**.603**	.231	.086	-.051
43	**.601**	.001	.095	.078
49	**.571**	-.110	.011	.080
27	**.570**	.066	.078	-.083
16	**.568**	.074	-.065	**-.323**
19	**.551**	-.058	.073	.172
12	**.527**	.109	.232	.130
38	**.519**	.191	-.124	.309
7	**.504**	.190	-.035	.061
3	**.501**	.119	-.077	.148
1	**.478**	.155	.016	-.008
45	**.437**	**.342**	.132	-.267
9	**.431**	.199	.066	.096
39	**.431**	.297	.192	-.115
33	**.413**	**.413**	-.031	-.154
18	**.401**	-.040	.154	.092
37	**.378**	.244	.278	-.138
32	**.341**	.233	.034	.251
	Factor 2			

Item	Factor			
	1	2	3	4
15	-.135	**.745**	-.059	.282
10	-.067	**.721**	.078	.170
53	.058	**.713**	.008	-.077
29	-.033	**.657**	.094	.033
47	.240	**.593**	-.004	-.004
13	.182	**.586**	-.019	-.066
54	.126	**.563**	.267	.082
40	.232	**.388**	.160	.050
50	.146	**.375**	-.037	**.431**
		Factor 3		
21	-.052	-.135	**.922**	-.005
51	.000	-.091	**.836**	.057
26	.151	.011	**.741**	-.024
23	-.122	.201	**.708**	.046
22	.127	-.034	**.607**	.006
20	.098	.314	**.442**	-.067
52	.272	.269	**.336**	-.053
		Factor 4		
6	.198	.014	-.177	**.692**
41	.303	-.129	.099	**.690**
31	.019	.019	.319	**.689**
8	.194	.127	-.114	**.673**
30	-.070	.242	.150	**.628**
25	-.143	.294	.306	**.481**
17	**.497**	-.245	.056	**.433**
4	.153	.235	.256	**.325**

Note. The present table refers to the final 46-item solution, imputed dataset 1. Factor loadings ≥ .32 are in bold.

Table S15

Factor Intercorrelations for the Four Factor Solution

Factor	1	2	3	4
1	—			
2	.400	—		
3	.319	.345	—	
4	.262	.129	.164	—

Note. The present table refers to the imputation dataset number 1.

Complete Case Exploratory Factor Analysis

The Complete Case 5-factor solution explained a total variance of 53.8%. The individual factor variance percentages were 15.1% (Factor 1), 11.9% (Factor 2), 10.1% (Factor 3), 8.5% (Factor 4), and 8.1% (Factor 5). Communalities ranged from .28 to .88 (M = .54; SD = .13) and are reported in Table S16. Factor loadings and compositions are reported from the pattern matrix for ease of interpretation in Table S17. Factor correlation analysis showed low-to-moderate intercorrelations (φ = .129 to .400; Table S18). The solution exhibited excellent reliability (ω = .955), with McDonald's ω for the single factors of .927 (Factor 1), .897 (Factor 2), .875 (Factor 3), .912 (Factor 4), and .846 (Factor 5).

Table S16

Item Communalities for the Complete Case Solution

Item	Communalities
1	.368
3	.411
4	.483
5	.632
6	.682
7	.396
8	.606
9	.398
10	.585
11	.552
12	.530
13	.549
15	.579
16	.390
17	.537

Item	Communalities
18	.279
19	.402
20	.419
21	.848
22	.392
23	.622
25	.695
26	.622
27	.376
28	.547
29	.498
30	.612
31	.629
33	.439
37	.458
38	.637
39	.493
40	.409
41	.614
42	.690
43	.481
44	.490
45	.519
46	.819
47	.517
48	.467
49	.339

Item	Communalities
50	.450
51	.736
52	.437
53	.658
54	.632
55	.884

Note. The present table refers to the 48-item solution.

Table S17

Pattern Matrix and Factor Composition for the Complete Case Solution

Item	Factor				
	1	2	3	4	5
			Factor 1		
16	**0,641**	-0,001	-0,054	0,040	-0,278
44	**0,615**	-0,100	-0,178	0,179	0,212
48	**0,609**	0,072	0,069	-0,016	0,073
28	**0,585**	0,256	0,067	0,021	-0,053
5	**0,584**	-0,199	0,242	0,137	0,260
11	**0,583**	0,075	0,270	-0,023	0,001
43	**0,580**	0,013	0,046	0,155	0,109
49	**0,549**	-0,065	-0,052	0,076	0,128
27	**0,545**	0,047	0,123	-0,005	-0,003
45	**0,515**	0,260	0,148	0,069	-0,253
38	**0,492**	0,153	-0,182	**0,415**	0,177
33	**0,460**	**0,370**	-0,036	0,025	-0,126
19	**0,459**	0,013	0,049	-0,005	0,309

Item	Factor				
	1	2	3	4	5
12	**0,450**	0,126	0,251	0,074	0,163
7	**0,448**	0,204	-0,031	-0,018	0,214
39	**0,436**	0,315	0,188	0,016	-0,126
3	**0,402**	0,149	-0,029	-0,055	**0,356**
1	**0,395**	0,189	0,093	-0,191	0,228
18	**0,349**	-0,056	0,176	0,030	0,222
37	**0,336**	0,300	0,297	-0,203	0,031
9	**0,333**	0,259	0,128	-0,137	0,248
Factor 2					
53	0,032	**0,778**	0,069	-0,249	0,045
15	-0,173	**0,737**	-0,038	0,132	0,202
10	-0,066	**0,733**	0,062	0,119	0,014
29	-0,019	**0,681**	0,042	0,107	-0,107
13	0,142	**0,649**	0,051	-0,284	0,082
47	0,266	**0,565**	-0,027	0,103	-0,037
54	0,127	**0,561**	0,206	0,234	-0,068
50	0,081	**0,448**	-0,086	0,240	0,275
40	0,239	**0,411**	0,128	0,118	-0,031
20	0,097	**0,354**	**0,378**	0,059	-0,107
Factor 3					
21	-0,113	-0,121	**0,982**	-0,014	-0,014
51	-0,100	-0,029	**0,892**	-0,074	0,085
26	0,147	0,033	**0,669**	0,180	-0,118
23	-0,117	0,199	**0,652**	0,247	-0,076
22	0,081	0,039	**0,568**	0,022	0,035
52	0,264	0,290	**0,341**	0,010	-0,056

Item	Factor				
	1	2	3	4	5
		Factor 4			
46	**0,423**	0,025	-0,090	**0,781**	-0,203
42	0,202	-0,215	0,299	**0,658**	0,041
25	-0,178	0,215	0,238	**0,656**	0,158
30	-0,147	0,235	0,046	**0,591**	0,307
55	0,267	-0,106	**0,327**	**0,576**	0,289
31	-0,101	0,043	0,216	**0,479**	**0,450**
4	0,113	0,285	0,131	**0,427**	0,093
		Factor 5			
6	-0,056	0,104	-0,087	0,008	**0,827**
8	0,019	0,179	-0,071	0,204	**0,659**
41	0,091	-0,030	0,089	0,204	**0,658**
17	0,303	-0,181	0,119	-0,042	**0,604**

Note. The present table refers to the 48-item solution. Factor loadings ≥ .32 are in bold.

Table S18

Factor Intercorrelations for the Four Factor Solution

Factor	1	2	3	4	5
1	—				
2	.370	—			
3	.309	.330	—		
4	.213	.171	.230	—	
5	.251	.135	.137	.245	—

Note. The present table refers to the 48-item solution.

Bifactorial Solution

Table S19

Schmid-Leiman loadings of single items on general and group factors – Imputation 1

Item	General	F1	F2	F3	F4	F5
1	.375	.352	.181	.034	-.072	.136
3	.393	.349	.131	-.028	-.012	.240
5	.512	.473	-.155	.178	.095	.280
6	.424	.375	.175	-.046	.072	.104
7	.423	.319	.228	.110	-.107	.205
8	.505	.488	.091	.196	.018	.000
9	.563	.383	.127	.205	.085	.156
10	.252	.500	.007	-.064	.049	-.241
11	.372	.285	-.012	.119	.115	.110
12	.417	.386	.001	.045	.052	.219
13	.407	.451	.064	.078	.005	.029
15	.503	.482	.226	.052	.021	-.001
16	.404	.355	.348	-.084	.116	-.163
17	.420	.317	.272	.224	-.089	-.032
18	.523	.354	.123	-.198	.345	.225
19	.476	.363	.289	.126	.046	-.080
20	.473	.459	.003	.061	.142	.070
21	.380	.448	-.072	-.159	.104	.302
22	.434	.400	.268	.050	.147	-.274
23	.460	.501	.072	.067	-.023	.121
25	.338	.420	-.071	-.013	.050	.142
26	.424	-.044	.611	.082	.069	.072
27	.328	.168	.567	.060	-.263	.073
28	.385	-.128	.635	-.035	.121	.157

Item	General	F1	F2	F3	F4	F5
29	.367	-.001	.565	.053	.067	-.040
30	.444	.195	.366	.076	.100	.006
31	.461	.210	.502	-.063	.158	-.077
33	.432	.048	.368	-.071	.140	.349
37	.338	.070	.672	.075	-.213	.029
38	.550	.122	.481	.181	.187	-.033
39	.422	.119	.302	.347	.047	-.095
40	.382	-.024	-.036	.827	-.012	-.008
41	.366	.124	.029	.484	.041	-.034
42	.444	-.071	.177	.556	.268	-.150
43	.495	.149	.057	.575	.166	-.108
44	.397	.001	.027	.779	-.097	.094
45	.305	-.008	.094	-.064	-.055	.795
46	.405	.025	.156	-.097	.165	.602
47	.377	.282	-.127	.114	-.077	.583
48	.488	-.098	.062	.184	.388	.444
49	.438	.103	-.015	.095	.084	.662
50	.493	-.185	.199	.149	.593	.149
51	.432	-.139	.178	.015	.467	.313
53	.486	.106	-.172	.197	.626	.025
54	.506	.222	.036	-.111	.703	-.131
55	.635	.185	-.094	.245	.495	.288

Table S20

Schmid-Leiman loadings of single items on general and group factors – Imputation 2

Item	General	F1	F2	F3	F4	F5
1	.366	.351	.176	.028	-.075	.140
3	.389	.342	.139	-.034	-.015	.247
5	.513	.480	-.158	.179	.096	.277
6	.309	-.001	.085	-.049	-.064	.781
7	.422	.371	.179	-.047	.065	.123
8	.405	.019	.159	-.102	.162	.603
9	.421	.320	.227	.109	-.103	.200
10	.427	-.047	.620	.092	.066	.062
11	.501	.496	.079	.195	.023	-.001
12	.561	.385	.122	.203	.086	.162
13	.326	.170	.569	.058	-.260	.066
15	.386	-.132	.641	-.036	.115	.160
16	.250	.500	.008	-.061	.049	-.231
17	.376	.277	-.122	.106	-.075	.583
18	.372	.282	-.010	.117	.112	.122
19	.414	.383	.004	.042	.042	.236
20	.422	.119	.302	.348	.041	-.084
21	.382	-.022	-.038	.826	-.010	-.014
22	.366	.119	.031	.483	.033	-.012
23	.446	-.072	.178	.558	.261	-.139
25	.496	-.188	.200	.150	.590	.150
26	.495	.148	.057	.577	.162	-.099
27	.405	.452	.063	.077	.012	.025
28	.501	.484	.223	.053	.024	.003
29	.366	.005	.561	.054	.067	-.045

Item	General	F1	F2	F3	F4	F5
30	.434	-.146	.182	.013	.463	.316
31	.489	-.103	.065	.180	.382	.447
33	.402	.358	.346	-.081	.121	-.168
37	.419	.317	.271	.224	-.092	-.020
38	.522	.353	.123	-.199	.346	.227
39	.475	.372	.283	.128	.051	-.087
40	.449	.202	.363	.082	.095	.015
41	.438	.099	-.011	.088	.082	.661
42	.487	.103	-.173	.200	.626	.026
43	.472	.455	.007	.061	.141	.080
44	.378	.446	-.073	-.161	.110	.300
45	.433	.405	.265	.053	.154	-.280
46	.507	.220	.034	-.107	.708	-.131
47	.460	.212	.501	-.061	.158	-.076
48	.458	.502	.073	.066	-.021	.122
49	.338	.423	-.071	-.013	.044	.157
50	.433	.050	.367	-.072	.136	.348
51	.398	.001	.027	.777	-.099	.096
53	.332	.073	.672	.069	-.213	.021
54	.551	.126	.479	.182	.184	-.030
55	.636	.182	-.093	.245	.494	.290

Table S21

Schmid-Leiman loadings of single items on general and group factors – Imputation 3

Item	General	F1	F2	F3	F4	F5
1	.375	.354	.179	.034	-.071	.138
3	.393	.347	.132	-.029	-.012	.242
5	.511	.473	-.157	.180	.093	.281
6	.304	-.009	.086	-.065	-.058	.794
7	.423	.371	.181	-.048	.073	.108
8	.405	.023	.154	-.097	.163	.602
9	.422	.322	.223	.110	-.108	.205
10	.432	-.038	.615	.100	.070	.053
11	.504	.492	.087	.197	.018	.002
12	.562	.385	.125	.205	.084	.158
13	.323	.167	.567	.053	-.265	.076
15	.392	-.133	.638	-.028	.134	.149
16	.250	.504	.003	-.062	.051	-.240
17	.377	.277	-.125	.113	-.078	.583
18	.373	.280	-.005	.117	.116	.113
19	.421	.384	.009	.046	.051	.222
20	.422	.119	.307	.343	.045	-.090
21	.384	-.025	-.033	.825	-.012	-.009
22	.366	.126	.027	.485	.040	-.035
23	.446	-.070	.180	.555	.267	-.148
25	.495	-.184	.198	.148	.592	.152
26	.496	.151	.058	.574	.164	-.106
27	.405	.454	.061	.078	.005	.030
28	.501	.487	.222	.053	.021	.000
29	.366	.003	.565	.049	.064	-.035

Item	General	F1	F2	F3	F4	F5
30	.432	-.138	.176	.016	.462	.315
31	.490	-.107	.072	.181	.385	.450
33	.403	.358	.349	-.086	.116	-.159
37	.420	.319	.273	.221	-.089	-.028
38	.521	.354	.122	-.199	.344	.230
39	.475	.368	.287	.124	.045	-.076
40	.448	.203	.363	.078	.097	.012
41	.438	.099	-.015	.094	.080	.665
42	.486	.107	-.174	.199	.627	.026
43	.472	.460	.002	.063	.142	.070
44	.377	.453	-.085	-.157	.104	.302
45	.433	.406	.264	.049	.148	-.271
46	.507	.220	.042	-.112	.704	-.126
47	.461	.212	.504	-.066	.158	-.072
48	.459	.501	.071	.067	-.023	.122
49	.337	.414	-.062	-.013	.048	.146
50	.431	.052	.361	-.072	.137	.352
51	.399	.001	.028	.777	-.098	.093
53	.338	.069	.679	.069	-.216	.034
54	.551	.125	.481	.178	.185	-.029
55	.635	.187	-.098	.247	.493	.289

Table S22

Schmid-Leiman loadings of single items on general and group factors - Imputation 4

Item	General	F1	F2	F3	F4	F5
1	.374	.353	.184	.033	-.074	.137
3	.390	.347	.136	-.032	-.012	.236
5	.512	.472	-.151	.180	.094	.280
6	.304	-.007	.088	-.065	-.056	.794
7	.423	.373	.180	-.046	.071	.106
8	.405	.022	.156	-.098	.164	.603
9	.423	.315	.232	.109	-.106	.205
10	.432	-.041	.613	.093	.074	.056
11	.504	.485	.096	.196	.019	.001
12	.562	.379	.132	.205	.086	.157
13	.328	.164	.570	.058	-.262	.074
15	.385	-.132	.634	-.037	.122	.157
16	.252	.502	.009	-.062	.050	-.242
17	.377	.282	-.125	.114	-.078	.583
18	.371	.291	-.013	.117	.115	.105
19	.417	.386	.007	.046	.048	.221
20	.422	.119	.303	.346	.046	-.093
21	.382	-.025	-.035	.826	-.012	-.008
22	.366	.123	.031	.484	.042	-.035
23	.446	-.070	.176	.555	.269	-.149
25	.494	-.187	.199	.147	.593	.152
26	.495	.148	.058	.575	.166	-.107
27	.405	.456	.063	.077	.007	.023
28	.502	.479	.231	.053	.022	.000
29	.367	-.003	.566	.051	.066	-.036

Item	General	F1	F2	F3	F4	F5
30	.433	-.142	.178	.014	.467	.315
31	.488	-.099	.061	.184	.385	.447
33	.399	.350	.349	-.087	.118	-.163
37	.420	.316	.275	.223	-.090	-.029
38	.522	.354	.124	-.198	.344	.228
39	.476	.363	.292	.125	.045	-.077
40	.448	.199	.363	.080	.097	.010
41	.438	.101	-.014	.095	.082	.664
42	.486	.107	-.171	.197	.627	.026
43	.473	.460	.005	.063	.143	.069
44	.378	.452	-.079	-.157	.104	.301
45	.434	.398	.272	.048	.148	-.271
46	.506	.223	.036	-.110	.703	-.129
47	.461	.207	.505	-.064	.158	-.074
48	.460	.503	.074	.068	-.024	.120
49	.338	.425	-.070	-.011	.047	.143
50	.431	.051	.359	-.071	.138	.351
51	.398	.000	.027	.779	-.097	.094
53	.338	.067	.675	.071	-.215	.032
54	.551	.120	.483	.179	.186	-.030
55	.635	.186	-.094	.246	.494	.288

Table S23

Schmid-Leiman loadings of single items on general and group factors – Imputation 5

Item	General	F1	F2	F3	F4	F5
1	.365	.348	.184	.029	-.078	.140
3	.371	.337	.134	-.040	-.017	.234
5	.513	.482	-.154	.180	.091	.279
6	.302	-.007	.085	-.070	-.063	.795
7	.422	.367	.188	-.045	.072	.108
8	.405	.022	.152	-.092	.157	.603
9	.420	.318	.229	.111	-.112	.208
10	.431	-.041	.610	.093	.073	.056
11	.502	.492	.089	.199	.017	.004
12	.560	.385	.126	.205	.083	.161
13	.325	.164	.570	.054	-.261	.074
15	.392	-.142	.636	-.024	.133	.150
16	.248	.503	.012	-.064	.062	-.247
17	.375	.282	-.123	.106	-.081	.583
18	.372	.278	.000	.121	.111	.116
19	.420	.384	.016	.049	.049	.221
20	.422	.115	.309	.342	.043	-.089
21	.384	-.024	-.035	.822	-.022	-.003
22	.366	.124	.026	.492	.030	-.028
23	.447	-.074	.177	.561	.254	-.138
25	.497	-.190	.195	.154	.585	.159
26	.496	.149	.059	.577	.157	-.101
27	.404	.452	.068	.079	.007	.029
28	.499	.483	.230	.053	.025	-.002
29	.366	-.003	.570	.044	.065	-.033

Item	General	F1	F2	F3	F4	F5
30	.434	-.142	.172	.023	.454	.321
31	.492	-.107	.068	.182	.376	.456
33	.400	.344	.352	-.065	.116	-.163
37	.418	.313	.280	.223	-.094	-.024
38	.522	.354	.126	-.197	.348	.226
39	.474	.365	.294	.123	.046	-.076
40	.448	.200	.365	.080	.097	.012
41	.438	.103	-.014	.087	.076	.665
42	.489	.105	-.173	.206	.620	.032
43	.470	.461	.005	.067	.142	.070
44	.376	.456	-.078	-.161	.111	.296
45	.432	.400	.272	.055	.149	-.269
46	.510	.216	.045	-.108	.712	-.127
47	.460	.203	.509	-.060	.159	-.071
48	.456	.502	.079	.062	-.018	.118
49	.339	.425	-.059	-.018	.049	.144
50	.431	.053	.358	-.074	.138	.349
51	.396	-.001	.027	.770	-.092	.076
53	.336	.067	.679	.065	-.217	.036
54	.551	.120	.484	.178	.183	-.026
55	.637	.187	-.099	.254	.485	.294

Table S24

General Factor Bifactor Indices Across Imputations

Index	Imputation				
	1	2	3	4	5
ECV	.356	.356	.356	.356	.356
ω_t	.968	.968	.968	.968	.968
ω_H	.591	.590	.591	.591	.590
H	.918	.918	.918	.918	.918
SO_{F1}	.654	.651	.650	.651	.647
SO_{F2}	.535	.533	.534	.535	.539
SO_{F3}	.485	.487	.486	.485	.491
SO_{F4}	.455	.459	.454	.455	.449
SO_{F5}	.273	.279	.287	.279	.288
SO_{var}	.246	.246	.247	.246	.247

Note. ECV = explained common variance; ω_t = Omega total; ω_H = omega Hierarchical; H = construct replicability index; SO_{Fx} = loading of factor x on the second-order factor; SO_{var} = proportion of variance explained by the second-order factor.

Table S25

Factor-level Bifactor Indices across imputations

Index	Factor				
	1	2	3	4	5
			Imputation 2		
ω_S	.944	.928	.923	.933	.930
ω_{HS}	.372	.407	.401	.411	.350
H	.820	.822	.841	.760	.815
			Imputation 3		

Index	Factor				
	1	2	3	4	5
ω_S	.944	.928	.915	.933	.930
ω_{HS}	.372	.407	.432	.412	.352
H	.820	.822	.839	.759	.821
			Imputation 4		
ω_S	.942	.928	.915	.932	.930
ω_{HS}	.382	.410	.433	.413	.350
H	.817	.822	.840	.759	.820
			Imputation 5		
ω_S	.942	.928	.922	.933	.930
ω_{HS}	.381	.413	.403	.404	.353
H	.818	.823	.838	.757	.821

Note. Factor level bifactor indices for imputation 1 are reported in the main text. ω_S = omega total for subscale; ω_{HS} = omega hierarchical for subscale; H = construct replicability index.

Reliability Analyses

Table S26

Reliability Coefficients Across Imputations for the Five Factor Solution

Factor	Imputation				
	1	2	3	4	5
Total	.952	.952	.952	.952	.952
1	.926	.926	.926	.926	.926
2	.894	.894	.895	.895	.895
3	.886	.886	.886	.886	.886
4	.909	.909	.909	.909	.909
5	.857	.855	.857	.857	.857

Note. Reliability was assessed using McDonald's omega computed from the polychoric correlation matrix

Table S27

Corrected Item-Total Correlations and Variations in Reliability Coefficients Across Imputations

Item	Imputation									
	1		2		3		4		5	
	ITC	Δω	ITC	Δω	ITC	Δω	ITC	Δω	ITC	Δω
					Factor 1					
1	.498	-.002	.489	-.002	.500	-.002	.499	-.002	.492	-.002
3	.385	-.003	.383	-.003	.386	-.003	.385	-.003	.374	-.002
**5**	**.290**	-.004	**.296**	-.005	**.290**	-.004	**.290**	-.004	**.293**	-.005
7	.378	-.003	.377	-.003	.378	-.003	.377	-.003	.377	-.003
9	.470	-.003	.468	-.003	.470	-.003	.469	-.003	.467	-.003
11	.625	-.006	.621	-.006	.626	-.006	.624	-.006	.621	-.006

Item	Imputation									
	1		2		3		4		5	
	ITC	Δω	ITC	Δω	ITC	Δω	ITC	Δω	ITC	Δω
12	.598	-.006	.595	-.006	.598	-.006	.595	-.006	.593	-.006
**16**	**.262**	-.001	**.262**	-.001	**.263**	-.001	**.264**	-.001	**.260**	-.001
18	.361	-.001	.360	-.001	.361	-.001	.364	-.001	.361	-.001
19	.421	-.003	.423	-.003	.429	-.003	.424	-.003	.427	-.003
27	.452	-.003	.450	-.003	.453	-.003	.454	-.003	.449	-.003
28	.613	-.006	.611	-.006	.613	-.006	.612	-.006	.610	-.006
33	.516	-.003	.514	-.003	.517	-.003	.509	-.003	.507	-.003
37	.558	-.003	.554	-.003	.558	-.003	.557	-.003	.555	-.003
**38**	**.206**	-.004	**.206**	-.004	**.206**	-.004	**.206**	-.004	**.206**	-.004
39	.543	-.004	.546	-.004	.543	-.004	.544	-.004	.545	-.004
43	.401	-.004	.400	-.004	.401	-.004	.402	-.004	.399	-.004
**44**	**.242**	-.002	**.240**	-.002	**.242**	-.002	**.241**	-.002	**.240**	-.003
45	.512	-.003	.510	-.003	.512	-.003	.511	-.003	.512	-.003
48	.353	-.005	.353	-.005	.353	-.005	.354	-.005	.351	-.005
49	.305	-.001	.313	-.001	.303	-.001	.309	-.001	.315	-.001
Factor 2										
10	.586	-.016	.593	-.016	.591	-.016	.586	-.016	.586	-.016
13	.564	-.010	.566	-.010	.563	-.010	.564	-.010	.565	-.010
15	.493	-.012	.494	-.012	.500	-.013	.493	-.012	.501	-.013
20	.428	-.004	.429	-.004	.431	-.004	.431	-.004	.431	-.004
29	.506	-.010	.505	-.010	.507	-.010	.507	-.010	.508	-.010
40	.477	-.006	.479	-.006	.478	-.006	.478	-.006	.478	-.006
47	.452	-.011	.451	-.011	.454	-.011	.453	-.011	.455	-.011
50	.331	-.004	.330	-.003	.326	-.003	.325	-.003	.325	-.003
53	.578	-.014	.577	-.014	.585	-.014	.580	-.014	.580	-.014

Item	Imputation									
	1		2		3		4		5	
	ITC	Δω	ITC	Δω	ITC	Δω	ITC	Δω	ITC	Δω
54	.643	-.017	.643	-.017	.646	-.017	.646	-.017	.647	-.017
Factor 3										
21	.694	-.041	.694	-.041	.694	-.041	.694	-.041	.693	-.040
22	.409	.001	.409	.001	.409	.001	.409	.001	.409	.001
23	.465	-.008	.465	-.008	.465	-.008	.465	-.008	.465	-.008
26	.599	-.027	.599	-.027	.599	-.027	.599	-.027	.599	-.027
51	.706	-.040	.706	-.040	.706	-.040	.706	-.040	.706	-.040
Factor 4										
25	.401	-.034	.401	-.034	.401	-.034	.401	-.034	.401	-.034
30	.346	-.008	.346	-.008	.346	-.008	.346	-.008	.346	-.008
31	.385	-.005	.385	-.005	.385	-.005	.385	-.005	.385	-.005
42	.490	-.019	.490	-.019	.490	-.019	.490	-.019	.490	-.019
46	.384	-.008	.384	-.008	.384	-.008	.384	-.008	.384	-.008
55	.443	-.023	.443	-.023	.443	-.023	.443	-.023	.443	-.023
Factor 5										
6	.514	-.078	.504	-.076	.514	-.078	.514	-.078	.514	-.078
8	.450	-.019	.446	-.019	.450	-.019	.450	-.019	.450	-.019
17	.333	.022	.329	.022	.333	.022	.333	.022	.333	.022
41	.493	-.049	.488	-.051	.493	-.049	.493	-.049	.493	-.049

Note. The present table refers to the final 46-item solution. Δω represents the variation in factor reliability after removal of the corresponding item. Item with ITC <.030 are presented in bold. ITC = corrected item-total correlation.

Receiver Operating Characteristic Analysis

Table S28

ROC Curve Characteristics for CDI Total Score and Subscales Across Imputations

Imputation	Optimal Youden's index	Sensitivity	Specificity	Cut-off
		Total Score		
1	.549	70.7%	84.1%	≥36
2	.549	70.7%	84.1%	≥36
3	.549	70.7%	84.1%	≥36
4	.549	70.7%	84.1%	≥36
5	.549	70.7%	84.1%	≥36
		Role Change and Adaptation to Disease		
1	.467	61.0%	85.7%	≥21
2	.467	61.0%	85.7%	≥21
3	.467	61.0%	85.7%	≥21
4	.467	61.0%	85.7%	≥21
5	.467	61.0%	85.7%	≥21
		Emotional Overwhelm		
1	.439	92.7%	51.2%	≥5
2	.435	92.7%	50.8%	≥5
3	.439	92.7%	51.2%	≥5
4	.439	92.7%	51.2%	≥5
5	.439	92.7%	51.2%	≥5
		Cognitive Challenges		
1	.355	75.6%	59.9%	≥1
2	.355	75.6%	59.9%	≥1
3	.355	75.6%	59.9%	≥1
4	.355	75.6%	59.9%	≥1
5	.355	75.6%	59.9%	≥1

Imputation	Optimal Youden's index	Sensitivity	Specificity	Cut-off
		Social Disconnection		
1	.397	48.8%	90.9%	≥2
2	.397	48.8%	90.9%	≥2
3	.397	48.8%	90.9%	≥2
4	.397	48.8%	90.9%	≥2
5	.397	48.8%	90.9%	≥2
		Health System Challenges		
1	.252	41.5%	83.7%	≥1
2	.276	43.9%	83.7%	≥1
3	.252	41.5%	83.7%	≥1
4	.252	41.5%	83.%	≥1
5	.252	41.5%	83.7%	≥1

Note. AUCs across imputations are reported as pooled results in the main text.

Table S29

ROC Curve Characteristics for CDI Total Score Across Imputations with PHQ-4 and Distress Thermometer as criteria

Imputation	Optimal Youden's index	Sensitivity	Specificity	Cut-off
		PHQ-4		
1	.552	82.4%	72.9%	≥24
2	.551	85.3%	69.8%	≥23
3	.551	85.3%	69.8%	≥23
4	.552	82.4%	72.9%	≥24
5	.552	82.4%	72.9%	≥24
		Distress Thermomether		
1	.400	.704	.696	≥21

Imputation	Optimal Youden's index	Sensitivity	Specificity	Cut-off
2	.407	.709	.698	≥21
3	.407	.709	.698	≥21
4	.407	.709	.698	≥21
5	.407	.709	.698	≥21

Note. AUCs across imputations are reported as pooled results in the main text.

Figure S1

ROC Curve comparison of K6, PHQ-4, and Distress Thermometer criteria

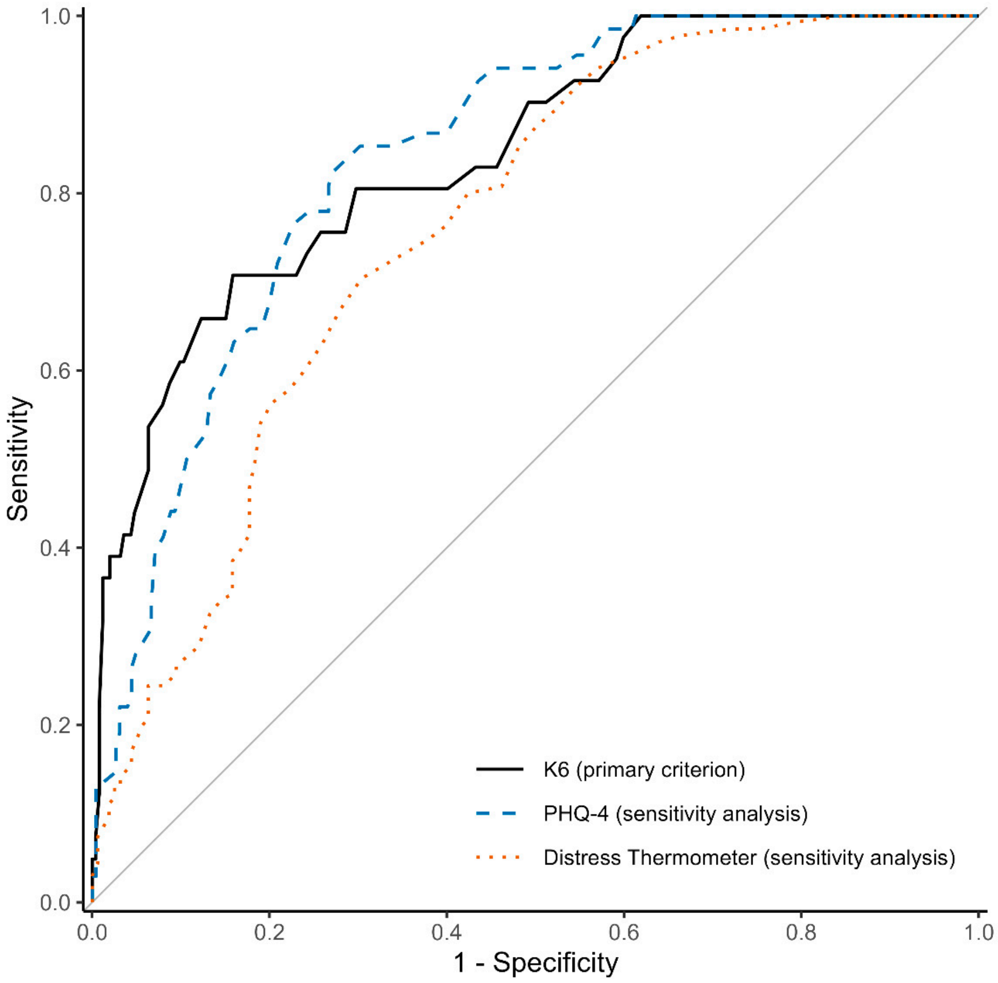

Note. Comparison of ROC Curves for CDI Total Score using K6, PHQ-4, and Distress Thermometer as Criteria. The present figures refer to imputation dataset number 1.

Figure S2

ROC Curve for Role Change and Adaptation to Disease

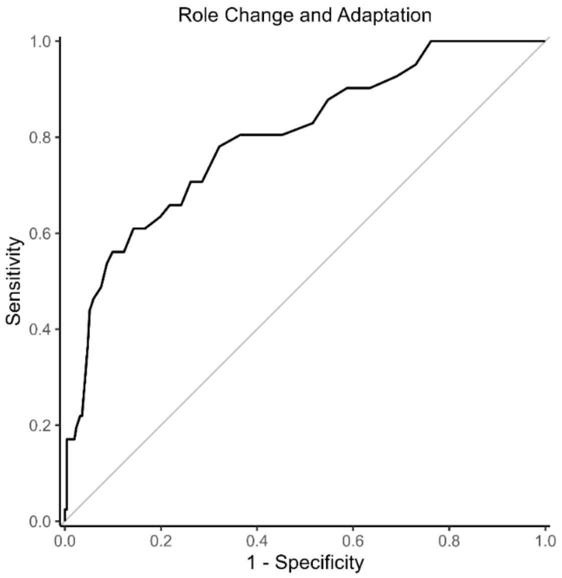

Note. ROC Curve for subscale Role Change and Adaptation to Disease using Kessler-6 as Criterion Standard. The present figures refer to imputation dataset number 1.

Figure S3

ROC Curve for Emotional Overwhelm

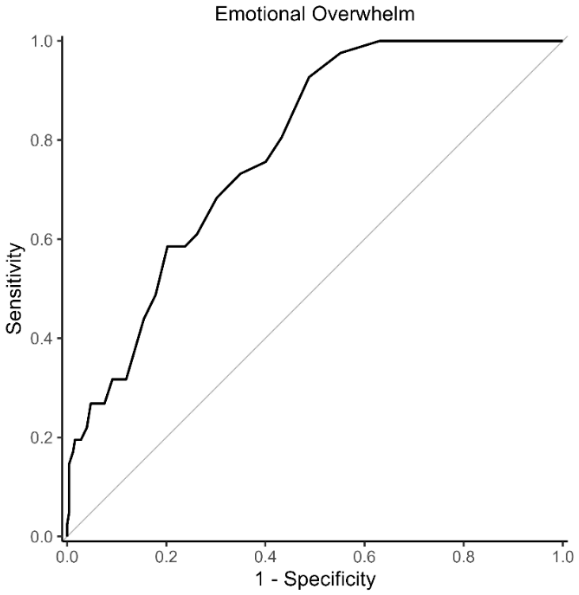

Note. ROC Curve for subscale Emotional Overwhelm using Kessler-6 as Criterion Standard. The present figures refer to imputation dataset number 1.

Figure S4

ROC Curve for Cognitive Challenges

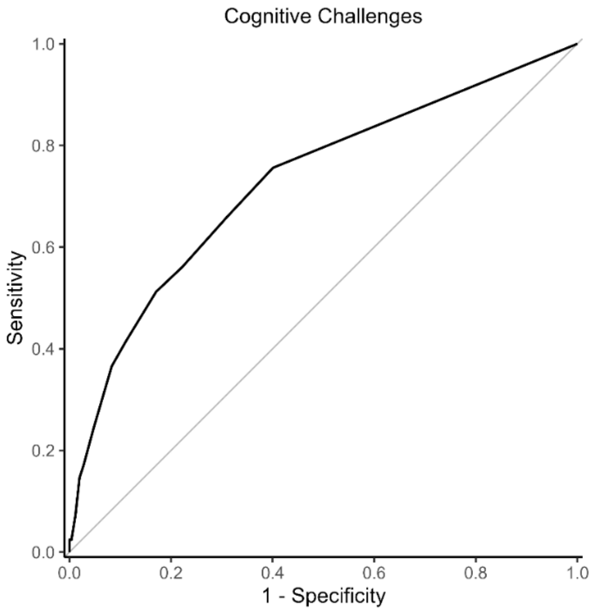

Note. ROC Curve for subscale Cognitive Challenges using Kessler-6 as Criterion Standard. The present figures refer to imputation dataset number 1.

Figure S5

ROC Curve for Social Disconnection

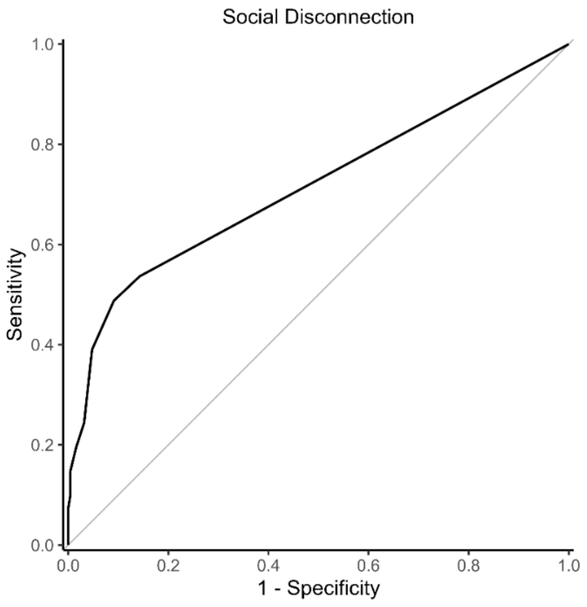

Note. ROC Curve for subscale Social Disconnection using Kessler-6 as Criterion Standard. The present figures refer to imputation dataset number 1.

Figure S6

ROC Curve for Health System Challenges

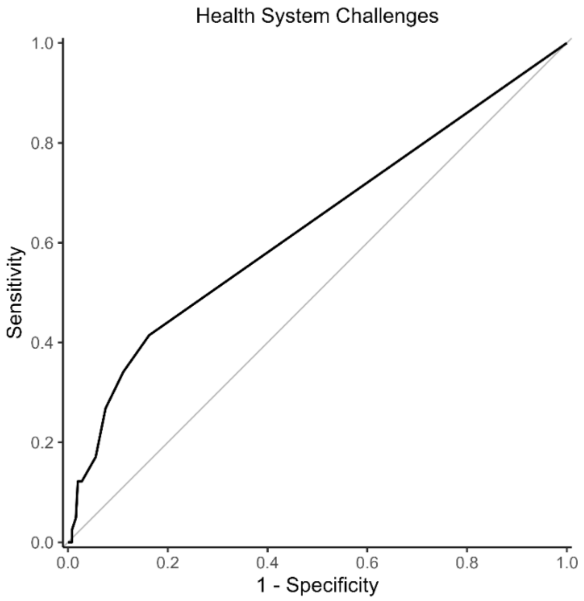

Note. ROC Curve for subscale Health System Challenges using Kessler-6 as Criterion Standard. The present figures refer to imputation dataset number 1.

Comparison with Original Questionnaire

The following items were removed from the questionnaire:

- Item 2: Being woken up at night by my racing heart;
- Item 4: Thinking my friends or family don't understand how difficult it is living with heart disease;
- Item 14: Being unable to accept help from others;
- Item 24: Being overly aware of my heart in my chest;
- Item 32: Thinking my condition might get worse;
- Item 34: Being unable to deal with stress;
- Item 35: Becoming a burden to my family;
- Item 36: Not knowing what the future holds for me;
- Item 52: Being short of breath.

Table S30

Item Distribution Across Factors: Comparison of Original and Italian CDI Structure

Factor	Role change and adaptation to disease	Emotional Overwhelm	Cognitive Challenges	Social Disconnection	Health System Challenges	Removed
Fear and Uncertainty	5 (62.5%)	1 (12.5%)	—	—	—	2 (25.0%)
Disconnection and Hopelessness	1 (12.5%)	—	—	5 (62.5%)	—	2 (25.0%)
Changes to Roles and Relationships	9 (81.8%)	—	—	1 (9.1%)	—	1 (9.1%)
Overwhelm and Depletion	3 (42.9%)	3 (42.9%)	—	—	—	1 (14.3%)
Cognitive Challenges	—	—	4 (100.0%)	—	—	—
Physical Challenges	3 (37.5%)	2 (25.0%)	—	—	—	3 (37.5%)
Health System Challenges	—	—	1 (20.0%)	—	4 (60.0%)	—
Death Concern	—	4 (100.0%)	—	—	—	—

Note. Heatmap comparing the eight-factor original CDI structure with the five-factor Italian structure. Numbers indicate how many items from each original factor (rows) loaded on each Italian factor (columns), with percentages shown in parentheses. Items that were removed (n = 9) are indicated in the rightmost column.
